# Baseline serum tumor markers predict the survival of patients with advanced non-small cell lung cancer receiving first-line immunotherapy: a multicenter retrospective study

**DOI:** 10.1186/s12885-023-11312-4

**Published:** 2023-08-30

**Authors:** Jian Huang, Yi Xiao, Yubin Zhou, Huiyin Deng, Zihao Yuan, Longyan Dong, Jun Lan, Xiane Li, Gaijiao Liu, Hao Hu, Shaohong Huang, Xiongwen Yang

**Affiliations:** 1 Department of Thoracic Surgery, Jiangxi Cancer Hospital, Nanchang, Jiangxi China; 2 Department of Cardio-Thoracic Surgery, Third Affiliated Hospital of Sun Yat-Sen University, Guangzhou, Guangdong China; 3 Department of Anesthesiology, Third Xiangya Hospital, Central South University, Changsha, Hunan China; 4 The Second Clinical Medical College, Guangdong Medical University, Dongguan, Guangdong China; 5 Department of General Surgery, the People’s Hospital of Gaoan City. Gaoan, Jiangxi, China; 6 Jiangxi Cancer Hospital, Nanchang, Jiangxi China; 7 Department of Anesthesiology, Shenzhen Second People’s Hospital, Guangzhou, China; 8 Department of Radiation Therapy, General Hospital of Southern Theater Command, Guangzhou, Guangdong China; 9 School of Medicine, South China University of Technology, Guangzhou, Guangdong China

**Keywords:** Non-small cell lung cancer, Baseline serum tumor markers, Immunotherapy, Progression-free survival, Overall survival

## Abstract

**Background:**

This study aimed to investigate the association between baseline serum tumor markers (STMs) (carcinoembryonic antigen [CEA], neuron-specific enolase [NSE], cytokeratin-19 fragment [CYFRA21-1], carbohydrate antigen 19–9 [CA19-9], and carbohydrate antigen 125 [CA125]) and the efficacy of first-line immunotherapy in patients with advanced non-small cell lung cancer.

**Methods:**

This multicenter retrospective study evaluated patients who received first-line immunotherapy between July 2017 and July 2022. The endpoints were progression-free survival (PFS) and overall survival (OS), as defined by the Response Evaluation Criteria in Solid Tumors version 1.1. We divided the patients into three groups based on STM levels: Group A ≥ threefold upper limit of normal, threefold upper limit of normal > Group B > upper limit of normal, and Group C ≤ upper limit of normal.

**Results:**

In total, 716 patients were included in this study. In Cox proportional hazards analyses, the STM levels in Group C were independently associated with superior PFS and OS in patients with lung adenocarcinoma (LUAD). Except for CA19-9 level, the STM levels in Group C were independently associated with superior PFS and OS in patients with lung squamous carcinoma (LUSC). Except for CEA and CA19-9 levels, the levels in Group A were independently associated with inferior PFS and OS in patients with LUAD and LUSC.

**Conclusions:**

Serum CEA, NSE, CYFRA21-1, and CA125 levels can predict PFS and OS in patients with LUAD and LUSC, and serum CA19-9 levels can predict PFS and OS in patients with LUAD. The higher the serum NSE, CYFRA21-1, and CA125 levels, the worse the PFS and OS in patients with LUAD and LUSC. In addition, the higher the serum CA19-9 level, the worse the OS in patients with LUAD.

**Supplementary Information:**

The online version contains supplementary material available at 10.1186/s12885-023-11312-4.

## Introduction

Lung cancer remains the main cause of death in patients with cancer, and the 5-year survival rate of patients with lung cancer is only approximately 15% [1, 2]. In the past, platinum-based chemotherapy has often been used to treat advanced lung cancer lacking the driving genes. With the advancement of immunotherapy in lung cancer, researchers have found that compared with traditional platinum-based chemotherapy strategies, immunotherapy can have better survival benefits to patients with advanced lung cancer who lack the driving genes [3–6]. Nevertheless, immunotherapy often fails because of tumor progression, and some patients do not benefit from immunotherapy [7]. Programmed cell death protein-1/programmed apoptosis ligand 1 (PD-L1) is the most widely known in the clinical application of immunotherapy for advanced lung cancer. The expression level of PD-L1 in patients with advanced lung cancer can be used to predict whether patients can benefit from immunotherapy [8, 9]. Nonetheless, the role of PD-L1 in predicting the prognosis of advanced lung cancer by immunotherapy is rather limited, with a single index, and the higher the expression of PD-L1, the better the prognosis of patients. Other common prediction methods include tumor mutational burden and circular tumor DNA, but these inevitably increase the extra cost of patients [10, 11].

Tumor targets are substances that exist in malignant tumor cells or are produced abnormally by malignant tumor cells. They can reflect the occurrence and development of tumors. Baseline levels of tumor markers, including carcinoembryonic antigen (CEA), cytokeratin-19 fragment (CYFRA 21–1), neuron-specific enolase (NSE), carbohydrate antigen 19–9 (CA19-9), and carbohydrate antigen 125 (CA125), have been proven to be associated with the prognosis of patients with advanced lung cancer receiving platinum-based chemotherapy [12–14]. However, the role of baseline tumor marker expression levels in predicting the prognosis of patients with advanced lung cancer receiving immunotherapy remains unknown.

This study included patients with advanced non-small cell lung cancer (NSCLC) from four cancer centers in China who received first-line immunotherapy. This study primarily aimed to evaluate the predictive effect of baseline serum tumor marker (STM) levels in patients with advanced NSCLC receiving first-line immunotherapy.

## Methods

### Study design

We retrospectively reviewed data from 716 patients with advanced NSCLC treated with first-line immunotherapy at the Third Affiliated Hospital of Sun Yat-sen University, General Hospital of Southern Theater Command, the Third Affiliated Xiangya Hospital of Central South University, and Jiangxi Cancer Hospital between July 2017 and July 2021.

### Inclusion criteria

The inclusion criteria were as follows: histologically confirmed NSCLC; diagnosis of stage IIIB to IV, including postoperative recurrence based on the eighth edition tumor-node-metastasis staging of the International Lung Cancer Research Association [15]; Eastern Cooperative Oncology Group performance status of 0–2; treatment with first-line immunotherapy; and adequate organ functions. The pathological diagnoses were performed according to the World Health Organization classification criteria [16].

Immunotherapy included immune checkpoint inhibitors with or without chemotherapy. The immune checkpoint inhibitors included pembrolizumab, nivolumab, atezolizumab, sintilimab, camrelizumab, and tirelizumab. Chemotherapy regimens included platinum-based regimens with or without bevacizumab. The duration of immunotherapy was at least 6 weeks.

Progression-free survival (PFS) was calculated from the initiation of treatment to definite tumor progression, death, or the last follow-up. Overall survival (OS) was calculated from the initiation of treatment to the date of death or last follow-up. All follow-up data were collected until October 31, 2022.

Tumor progression was assessed using the Response Evaluation Criteria in Solid Tumors version 1.1 [17], including complete response (CR), partial response (PR), stable disease (SD), and progressive disease (PD). Objective response rate (ORR) was defined as the percentage of CR + PR after immunotherapy. Disease control rate (DCR) was defined as the percentage of CR + PR + SD after immunotherapy. Efficacy was evaluated independently by two experienced physicians. Considering the possibility of pseudoprogression in immunotherapy, determination of disease progression requires two consecutive radiological examinations.

STM (CEA, NSE, CYFRA 21–1, CA19-9, and CA125) concentrations were measured at the time of diagnosis. For the reported cohort, STM analyses were performed using a cobas e 801 immunoassay module (Roche Diagnostics, Rotkreuz, Switzerland) and the corresponding ElectroChemiLuminescence-ImmunoAssay kits acquired from Roche. According to the manufacturer’s instructions, the normal upper limits of CEA, NSE, CYFRA 21–1, CA19-9, and CA125 for the diagnosis of NSCLC are 5.00, 16.3, 3.30, 27.0, and 35.0 ng/mL, respectively.

We divided the patients into three groups according to the baseline STM levels. Group A had baseline STM levels greater than three times the upper limit of the normal value. In Group B, the baseline STM levels were higher than the upper limit of the normal value and less than three times the upper limit of the normal value. In Group C, the baseline STM levels were lower than the upper limit of normal.

### Statistical analyses

Continuous variables are presented as mean ± standard deviation, and categorical variables are presented as numbers (%). Categorical variables were compared using the chi-squared or Fisher’s exact test. Survival was estimated using the Kaplan–Meier method. A log-rank test was performed to evaluate the significance of the differences in survival periods among the groups. The median, 95% confidence intervals (CIs), and *P* values from the log-rank tests are reported in the figures. The Cox proportional hazards regression model was used for univariate and multivariate analyses to assess the prognostic role of STMs, adjusted for the possible confounding effect of all other factors included in the same model. All *P* values were two-sided, and values < 0.05 were considered statistically significant. Statistical analyses were performed using the R software version 4.2.1 (https://www.r-project.org/).

## Results

### Patient selection and characteristics

**Fig. 1:**
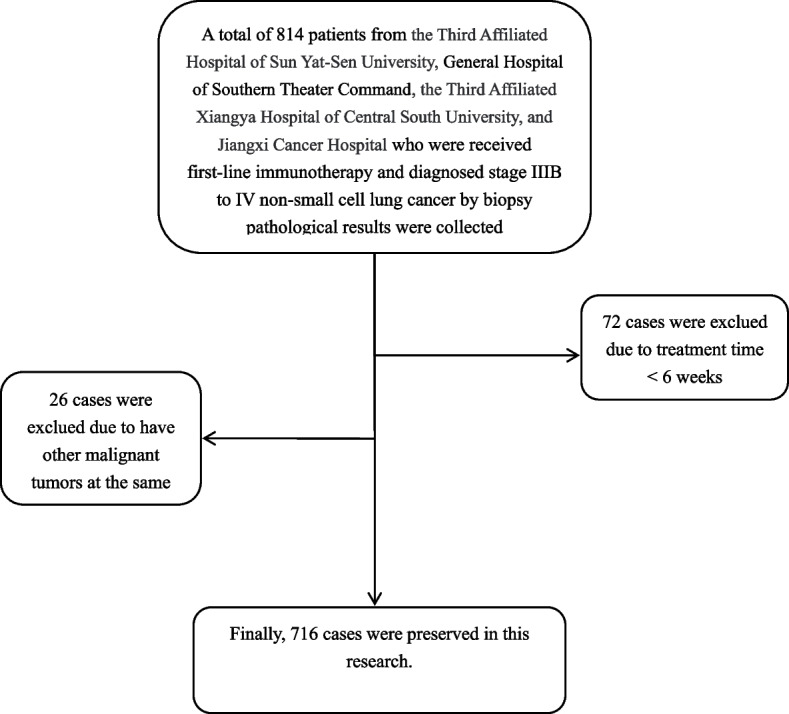
Flow-chart of this study

The flow diagram of the patients included in the analysis is shown in Fig. 1. Clinicopathological characteristics of the patients are shown in Table 1. In total, 716 patients with advanced NSCLC were included in this study, including 390 patients with lung adenocarcinoma (LUAD), 280 with lung squamous carcinoma (LUSC), and 46 with other types of NSCLC. The mean age of the included patients was 61.1 years, and the majority of the patients were male. In total, 605 patients had stage IV disease. Smoking and previous smoking accounted for the majority of the patients. Only a small proportion of patients were negative for PD-L1 expression. Most patients received immune checkpoint blockades in combination with chemotherapy. The mean CEA level was 57.29 ng/mL, and 409 (57.1%) patients had CEA levels above the upper limit of normal. The mean NSE level was 24.26 ng/mL, and 414 (57.8%) patients had NSE levels above the upper limit of normal. The mean CYFRA21-1 level was 15.69 ng/mL, and 618 (86.3%) patients had CYFRA21-1 levels above the upper limit of normal. The mean CA19-9 level was 45.99 ng/mL, and 268 (37.4%) patients had CA19-9 levels above the upper limit of normal. The mean CA125 level was 69.04 ng/mL, and 416 (58.1%) patients had CA125 levels above the upper limit of normal.

**Table 1:** Characteristics of patients at baseline

Characteristics	Patients (*n* = 716)	Percentage (%)
**Age** (Mean ± SD)	61.10 ± 10.55	
**Sex**		
Male	611	85.3
Female	105	14.8
**Histological type**		
LUAD	390	54.5
LUSC	280	39.1
Other NSCLC	46	6.4
**Clinical stage**		
IIIB	86	12
IIIC	25	3.5
IV	605	84.5
**Smoking history**		
Never smoker	324	45.3
Smoker or ex-smoker	392	54.7
**PD-L1 expression**		
< 1%	154	21.5
1%-49%	298	41.6
≥ 50%	264	36.9
**Treatment type**		
Monotherapy	284	39.7
Combination therapy	432	60.3
**ECOG PS**		
0–1	645	90.1
2	71	9.9
**Radiation history**		
Yes	452	63
No	265	37
**Metastasis sites**		
Liver	59	8.24
Lung	179	25
Brain	135	18.9
Bone	213	29.7
Adrenal	124	17.3
**Drug**		
Pembrolizumab	451	63
Nivolumab	108	15.1
Atezolizumab	5	0.7
Sintilimab	108	15.1
Camrelizumab	31	4.3
Tislelizumab	13	1.8
**CEA (ng/ml)**		
Mean ± SD	57.29 ± 159.72	
Normal (≤ 5.0)	307	42.9
High (> 5.0)	409	57.1
**NSE (ng/ml)**		
Mean ± SD	24.26 ± 18.19	
Normal (≤ 16.3)	302	42.2
High (> 16.3)	414	57.8
**CYFRA21-1 (ng/ml)**		
Mean ± SD	15.69 ± 27.37	
Normal (≤ 3.3)	98	13.7
High (> 3.3)	618	86.3
**CA19-9 (ng/ml)**		
Mean ± SD	45.99 ± 106.47	
Normal (≤ 27.0)	448	62.6
High (> 27.0)	268	37.4
**CA125 (ng/ml)**		
Mean ± SD	69.04 ± 112.97	
Normal (≤ 35.0)	300	41.9
High (> 35.0)	416	58.1

*LUAD* Lung adenocarcinoma, *LUSC* Lung squamous cell carcinoma, *NSCLC* Non-small cell lung cancer, *PD-L1* Programmed death ligand-1, *CEA* Carcinoembryonic antigen, *NSE* Neuron-specific enolase, *CYFRA21-1* Cytokeratin fragment 19, *CA19-9* Carbohydrate antigen 19–9, *CA125* Carbohydrate antigen 125, *ECOG PS* Eastern cooperative oncology group performance status

### Association between baseline serum tumor marker (STM) levels and survival

#### Analysis of the whole population

**Fig. 2:**
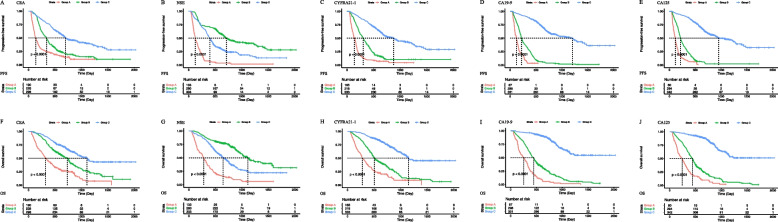
Kaplan–Meier curves of progression-free survival and overall survival in whole population. Kaplan–Meier curves are based on baseline CEA (A and F), NSE (B and G), CYFRA21-1 (C and H), CA19-9 (D and I), and CA125 (E and J) levels Group A: Baseline STM levels greater than three times the upper limit of the normal value. Group B: Baseline STM levels were higher than the upper limit of the normal value and less than three times the upper limit of the normal value. Group C: Baseline STM levels were lower than the upper limit of normal

The median PFS and OS periods of the 716 patients were 398 days (95% CI, 352–540 days) and 418 days (95% CI, 678–797 days), respectively. By univariate analysis, CEA (Group A vs. Group C, HR [95% CI] = 0.53 [0.44–0.66], *P* < 0.001), NSE (Group A vs. Group B, HR [95% CI] = 0.40 [0.29–0.55], *P* < 0.001; Group A vs. Group C, HR [95% CI] = 0.27 [0.19–0.38], *P* < 0.001), CYFRA21-1 (Group A vs. Group B, HR [95% CI] = 0.72 [0.60–0.86], *P* < 0.001; Group A vs. Group C, HR [95% CI] = 0.49 [0.36–0.66], *P* < 0.001), CA19-9 (Group A vs. Group C, HR [95% CI] = 0.56 [0.41–0.77], *P* < 0.001), and CA125 (Group A vs. Group B, HR [95% CI] = 0.59 [0.46–0.77], *P* < 0.001; Group A vs. Group C, HR [95% CI] = 0.48 [0.37–0.63], *P* < 0.001) levels were associated with significantly different PFS among subgroups (Table 2, Fig. 2). Similarly, CEA (Group A vs. Group C, HR [95% CI] = 0.50 [0.40–0.63], *P* < 0.001), NSE (Group A vs. Group B, HR [95% CI] = 0.38 [0.27–0.53], *P* < 0.001; Group A vs. Group C, HR [95% CI] = 0.25 [0.17–0.35], *P* < 0.001), CYFRA21-1 (Group A vs. Group B, HR [95% CI] = 0.66 [0.54–0.80], *P* < 0.001; Group A vs. Group C, HR [95% CI] = 0.39 [0.27–0.55], *P* < 0.001), CA19-9 (Group A vs. Group B, HR [95% CI] = 0.62 [0.44–0.88], *P* = 0.007; Group A vs. Group C, HR [95% CI] = 0.45 [0.32–0.62], *P* < 0.001), and CA125 (Group A vs. Group B, HR [95% CI] = 0.56 [0.42–0.74], *P* < 0.001; Group A vs. Group C, HR [95% CI] = 0.44 [0.33–0.59], *P* < 0.001) levels were associated with significantly different OS among subgroups (Table 2, Fig. 2).

**Table 2:** Prognostic factors for progression-free survival and overall survival in patients with advanced non-small cell lung cancer

Covariate	Univariate analysis		Multivariate analysis (CEA)		Multivariate analysis (NSE)		Multivariate analysis (CYFRA21-1)		Multivariate analysis (CA19-9)		Multivariate analysis (CA125)	
	*P*	HR (95% CI)	*P*	HR (95% CI)	*P*	HR (95% CI)	*P*	HR (95% CI)	*P*	HR (95% CI)	*P*	HR (95% CI)
**Progression-free survival**												
Age	0.692		0.398		0.367		0.251		0.148		0.167	
Old (≥ 65)		1		1		1		1		1		1
Young (< 65)		0.97(0.81–1.15)		0.92(0.77–1.11)		0.92(0.77–1.10)		0.90(0.75–1.08)		0.87(0.73–1.05)		0.88(0.73–1.06)
Sex	0.732		0.33		0.025		0.226		0.109		0.137	
Female		1		1		1		1		1		1
Male		1.05(0.81–1.34)		1.15(0.87–1.52)		1.38(1.04–1.82)		1.19(0.90–1.57)		1.25(0.95–1.65)		1.23(0.94–1.62)
ECOG PS	< 0.001		< 0.001		< 0.001		< 0.001		< 0.001		< 0.001	
0–1		1		1		1		1		1		1
2		3.06(2.36–3.97)		2.75(2.10–3.60)		2.96(2.26–3.88)		2.98(2.27–3.91)		2.94(2.24–3.86)		3.19(2.43–4.19)
Smoke history	0.191		0.01		0.214		0.154		0.106		0.039	
No		1		1		1		1		1		1
Yes		0.89(0.75–1.06)		0.77(0.63–0.94)		0.88(0.72–1.07)		0.87(0.71–1.06)		0.85(0.70–1.04)		0.81( 0.67–0.99)
Histological type	< 0.001		< 0.001		< 0.001		0.007		0.001		< 0.001	
LUAD		1		1		1		1		1		1
LUSC	< 0.001	1.37(1.14–1.63)	< 0.001	1.57(1.29–1.90)	< 0.001	1.37(1.14–1.65)	0.018	1.26(1.04–1.53)	0.001	1.35(1.12–1.63)	< 0.001	1.43(1.18–1.72)
Other_NSCLC	0.003	0.50(0.32–0.79)	0.006	0.53(0.33–0.84)	< 0.001	0.45(0.29–0.71)	0.005	0.52(0.33–0.82)	0.001	0.47(0.30–0.74)	0.004	0.51(0.32–0.80)
Stage	0.002		< 0.001		0.005		0.008		0.01		0.002	
IIIB		1		1		1		1		1		1
IIIC	< 0.001	2.67(1.63–4.37)	< 0.001	2.52(1.51–4.23)	0.003	2.20(1.31–3.70)	0.005	2.24(1.33–3.76)	0.003	2.21(1.32–3.72)	0.001	2.32(1.38–3.91)
IV	0.134	1.24(0.94–1.64)	0.079	1.30(0.97–1.74)	0.07	1.31(0.98–1.76)	0.042	1.09(0.82–1.47)	0.07	1.31(0.98–1.76)	0.065	1.32(0.98–1.77)
PD-L1 expression	0.048		0.316		0.481		0.376		0.208		0.19	
< 1%		1		1		1		1		1		1
≥ 1%		0.81(0.66–1.00)		0.89(0.71–1.11)		0.92(0.74–1.15)		0.90(0.72–1.13)		0.87(0.70–1.08)		0.86(0.69–1.08)
Treatment	0.133		0.022		0.038		0.024		0.133		0.03	
Monotherapy		1		1		1		1		1		1
Combination therapy		0.87(0.73–1.04)		0.80(0.66–0.97)		0.82(0.67–0.99)		0.80(0.66–0.97)		0.86(0.71–1.05)		0.81(0.67–0.98)
CEA	< 0.001		< 0.001									
3-Fold(> 15)		1		1								
Normal to 3-Fold (5–15)	0.163	0.86(0.69–1.07)	0.043	0.79(0.63–0.99)								
Normal (≤ 5.0)	< 0.001	0.53(0.44–0.66)	< 0.001	0.45(0.36–0.56)								
NSE	< 0.001				< 0.001							
3-Fold(> 48.9)		1				1						
Normal to 3-Fold (16.3–48.9)	< 0.001	0.40(0.29–0.55)			< 0.001	0.39(0.28–0.54)						
Normal (≤ 16.3)	< 0.001	0.27(0.19–0.38)			< 0.001	0.26(0.18–0.36)						
CYFRA21-1	< 0.001						< 0.001					
3-Fold(> 9.9)		1						1				
Normal to 3-Fold (3.3–9.9)	< 0.001	0.72(0.60–0.86)					0.014	0.79(0.65–0.95)				
Normal (≤ 3.3)	< 0.001	0.49(0.36–0.66)					< 0.001	0.56(0.42–0.77)				
CA19-9	< 0.001								< 0.001			
3-Fold(> 81)		1								1		
Normal to 3-Fold (27–81)	0.07	0.74(0.53–1.03)							0.063	0.73(0.52–1.02)		
Normal (≤ 27.0)	< 0.001	0.56(0.41–0.77)							< 0.001	0.56(0.41–0.76)		
CA125	< 0.001										< 0.001	
3-Fold(> 105)		1										1
Normal to 3-Fold (35–105)	< 0.001	0.59(0.46–0.77)									< 0.001	0.49(0.37–0.64)
Normal (≤ 35.0)	< 0.001	0.48(0.37–0.63)									< 0.001	0.39(0.30–0.53)
**Overall survival**												
Age	0.023		0.013		0.018		0.005		0.001		0.002	
Old (≥ 65)		1		1		1		1		1		1
Young (< 65)		0.80(0.66–0.97)		0.77(0.63–0.95)		0.78(0.64–0.96)		0.75(0.61–0.92)		0.72(0.59–0.89)		0.73(0.59–0.89)
Sex	0.092		0.115		0.041		0.078		0.035		0.06	
Female		1		1		1		1		1		1
Male		1.28(0.96–1.70)		1.29(0.94–1.78)		1.39(1.01–1.92)		1.33(0.97–1.83)		1.41(1.02–1.93)		1.36(0.99–1.86)
ECOG PS	< 0.001		< 0.001		< 0.001		< 0.001		< 0.001		< 0.001	
0–1		1		1		1		1		1		1
2		3.22(2.46–4.21)		3.09(2.33–4.08)		3.25(2.45–4.31)		3.31(2.50–4.40)		3.28(2.47–4.35)		3.49(2.62–4.63)
Smoke history	0.384		0.484		0.404		0.984		0.767		0.806	
No		1		1		1		1		1		1
Yes		1.09(0.90–1.32)		0.93(0.75–1.15)		1.10(0.88–1.36)		1.00(0.81–1.24)		0.97(0.78–1.20)		0.97(0.78–1.21)
Histological type	< 0.001		< 0.001		0.005		0.041		0.001		< 0.001	
LUAD		1		1		1		1		1		1
LUSC	0.002	1.36(1.12–1.66)	< 0.001	1.70(1.37–2.11)	0.001	1.40(1.14–1.72)	0.034	1.26(1.02–1.55)	0.001	1.41(1.14–1.73)	< 0.001	1.49(1.21–1.83)
Other_NSCLC	0.042	0.59(0.36–0.98)	0.073	0.62(0.37–1.04)	0.019	0.54(0.32–0.90)	0.085	0.64(0.38–1.06)	0.002	0.55(0.33–0.92)	0.054	0.60(0.36–1.01)
Stage	0.034		0.042		0.234		0.159		0.162		0.068	
IIIB		1		1		1		1		1		1
IIIC	0.006	2.10(1.23–3.57)	0.014	2.00(1.15–3.46)	0.187	1.45(0.83–2.53)	0.121	1.55(0.89–2.71)	0.075	1.66(0.95–2.90)	0.043	1.78(1.02–3.10)
IV	0.3	1.18(0.86–1.62)	0.252	062(0.37–1.04)	0.276	1.20(0.86–1.67)	0.187	1.25(0.90–1.74)	0.265	1.21(0.87–1.68)	0.235	1.22(0.88–3.10)
PD-L1 expression	0.001		0.265		0.209		0.172		0.087		0.044	
< 1%		1		1		1		1		1		1
≥ 1%		0.69(0.55–0.87)		0.87(0.69–1.11)		0.86(0.67–1.09)		0.85(0.67–1.07)		0.81(0.64–1.03)		0.78(0.62–0.99)
Treatment	0.103		0.013		0.044		0.012		0.107		0.023	
Monotherapy		1		1		1		1		1		1
Combination therapy		0.85(0.70–1.03)		0.76(0.62–0.94)		0.80(0.65–0.99)		0.76(0.62–0.94)		0.84(0.68–1.04)		0.78(0.63–0.97)
CEA	< 0.001		< 0.001									
3-Fold(> 15)		1		1								
Normal to 3-Fold (5–15)	0.539	0.93(0.73–1.18)	0.139	0.83(0.65–1.06)								
Normal (≤ 5.0)	< 0.001	0.50(0.40–0.63)	< 0.001	0.42(0.32–0.54)								
NSE	< 0.001				< 0.001							
3-Fold(> 48.9)		1				1						
Normal to 3-Fold (16.3–48.9)	< 0.001	0.38(0.27–0.53)			< 0.001	0.40(0.29–0.56)						
Normal (≤ 16.3)	< 0.001	0.25(0.17–0.35)			< 0.001	0.26(0.18–0.37)						
CYFRA21-1	< 0.001						< 0.001					
3-Fold(> 9.9)		1						1				
Normal to 3-Fold (3.3–9.9)	< 0.001	0.66(0.54–0.80)					< 0.001	0.69(0.56–0.85)				
Normal (≤ 3.3)	< 0.001	0.39(0.27–0.55)					< 0.001	0.45(0.31–0.64)				
CA19-9	< 0.001								< 0.001			
3-Flod(> 81)		1								1		
Normal to 3-Fold (27–81)	0.007	0.62(0.44–0.88)							0.001	0.55(0.38–0.79)		
Normal (≤ 27.0)	< 0.001	0.45(0.32–0.62)							< 0.001	0.41(0.30–0.58)		
CA125	< 0.001										< 0.001	
3-Fold(> 105)		1										1
Normal to 3-Fold (35–105)	< 0.001	0.56(0.42–0.74)									< 0.001	0.44(0.33–0.59)
Normal (≤ 35.0)	< 0.001	0.44(0.33–0.59)									< 0.001	0.35(0.26–0.48)

*ECOG PS* Eastern cooperative oncology group performance status, *PD-L1* Programmed death ligand-1, *CEA* Carcinoembryonic antigen, *NSE* Neuron-specific enolase, *CYFRA21-1* Cytokeratin fragment 19, *CA19-9* Carbohydrate antigen 19–9, *CA125* Carbohydrate antigen 125

By multivariate analysis, CEA (Group A vs. Group B, HR [95% CI] = 0.79 [0.63–0.99], *P* = 0.043; Group A vs. Group C, HR [95% CI] = 0.45 [0.36–0.56], *P* < 0.001), NSE (Group A vs. Group B, HR [95% CI] = 0.39 [0.28–0.54], *P* < 0.001; Group A vs. Group C, HR [95% CI] = 0.26 [0.18–0.36], *P* < 0.001), CYFRA21-1 (Group A vs. Group B, HR [95% CI] = 0.79 [0.65–0.95], *P* = 0.014; Group A vs. Group C, HR [95% CI] = 0.56 [0.42–0.77], *P* < 0.001), CA19-9 (Group A vs. Group C, HR [95% CI] = 0.56 [0.41–0.76], *P* < 0.001), and CA125 (Group A vs. Group B, HR [95% CI] = 0.49 [0.37–0.64], *P* < 0.001; Group A vs. Group C, HR [95% CI] = 0.39 [0.30–0.53], *P* < 0.001) levels were associated with significantly different PFS among subgroups (Table 2). Similarly, by multivariate analysis, CEA (Group A vs. Group C, HR [95% CI] = 0.42 [0.32–0.54], *P* < 0.001), NSE (Group A vs. Group B, HR [95% CI] = 0.40 [0.29–0.56], *P* < 0.001; Group A vs. Group C, HR [95% CI] = 0.26 [0.18–0.37], *P* < 0.001), CYFRA21-1 (Group A vs. Group B, HR [95% CI] = 0.69 [0.56–0.85], *P* < 0.001; Group A vs. Group C, HR [95% CI] = 0.45 [0.31–0.64], *P* < 0.001), CA19-9 (Group A vs. Group B, HR [95% CI] = 0.55 [0.38–0.79], *P* = 0.001; Group A vs. Group C, HR [95% CI] = 0.41 [0.30–0.58], *P* < 0.001), and CA125 (Group A vs. Group B, HR [95% CI] = 0.44 [0.33–0.59], *P* < 0.001; Group A vs. Group C, HR [95% CI] = 0.35 [0.26–0.48], *P* < 0.001) levels were associated with significantly different OS among subgroups (Table 2).

### Analysis of patients with lung adenocarcinoma

**Fig. 3:**
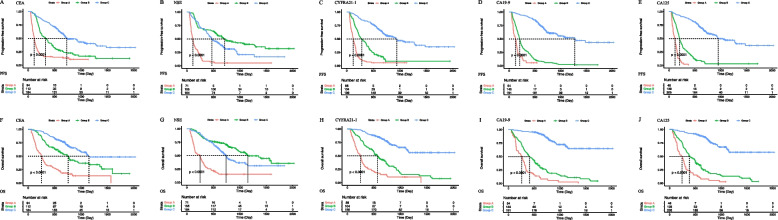
Kaplan–Meier curves of progression-free survival and overall survival in LUAD population. Kaplan–Meier curves are based on baseline CEA (A and F), NSE (B and G), CYFRA21-1 (C and H), CA19-9 (D and I), and CA125 (E and J) Group A: Baseline STM levels greater than three times the upper limit of the normal value. Group B: Baseline STM levels were higher than the upper limit of the normal value and less than three times the upper limit of the normal value. Group C: Baseline STM levels were lower than the upper limit of normal

Overall, the median PFS and OS periods of the 390 patients with LUAD were 446 days (95% CI, 372–528 days) and 760 days (95% CI, 704–931 days), respectively. By univariate analysis, CEA (Group A vs. Group C, HR [95% CI] = 0.38 [0.28–0.52], *P* < 0.001), NSE (Group A vs. Group B, HR [95% CI] = 0.37 [0.24–0.58], *P* < 0.001; Group A vs. Group C, HR [95% CI] = 0.24 [0.15–0.39], *P* < 0.001), CYFRA21-1 (Group A vs. Group B, HR [95% CI] = 0.75 [0.58–0.98], *P* = 0.033; Group A vs. Group C, HR [95% CI] = 0.57 [0.37–0.86], *P* = 0.007), CA19-9 (Group A vs. Group B, HR [95% CI] = 0.67 [0.44–0.99], *P* = 0.042; Group A vs. Group C, HR [95% CI] = 0.43 [0.30–0.64], *P* < 0.001), and CA125 (Group A vs. Group B, HR [95% CI] = 0.70 [0.51–0.96], *P* = 0.026; Group A vs. Group C, HR [95% CI] = 0.41 [0.29–0.59], *P* < 0.001) levels in patients with LUAD were associated with significantly different PFS among subgroups (Table 3, Fig. 3). Similarly, CEA (Group A vs. Group C, HR [95% CI] = 0.33 [0.23–0.48], *P* < 0.001), NSE (Group A vs. Group B, HR [95% CI] = 0.37 [0.24–0.59], *P* < 0.001; Group A vs. Group C, HR [95% CI] = 0.22 [0.14–0.36], *P* < 0.001), CYFRA21-1 (Group A vs. Group B, HR [95% CI] = 0.69 [0.52–0.92], *P* = 0.012; Group A vs. Group C, HR [95% CI] = 0.38 [0.23–0.64], *P* < 0.001), CA19-9 (Group A vs. Group B, HR [95% CI] = 0.54 [0.35–0.82], *P* = 0.004; Group A vs. Group C, HR [95% CI] = 0.33 [0.22–0.49], *P* < 0.001), and CA125 (Group A vs. Group B, HR [95% CI] = 0.62 [0.43–0.88], *P* < 0.001; Group A vs. Group C, HR [95% CI] = 0.38 [0.25–0.56], *P* < 0.001) levels in patients with LUAD were associated with significantly different OS among subgroups (Table 3, Fig. 3).

**Table 3:** Prognostic factors for progression-free survival and overall survival in patients with lung adenocarcinoma

Covariate	Univariate analysis		Multivariate analysis (CEA)		Multivariate analysis (NSE)		Multivariate analysis (CYFRA21-1)		Multivariate analysis (CA19-9)		Multivariate analysis (CA125)	
	*P*	HR (95% CI)	*P*	HR (95% CI)	*P*	HR (95% CI)	*P*	HR (95% CI)	*P*	HR (95% CI)	*P*	HR (95% CI)
**Progression-free survival**												
Age	0.458		0.415		0.251		0.277		0.057		0.106	
Old (≥ 65)		1		1		1		1		1		1
Young (< 65)		0.91(0.72–1.16)		0.90(0.70–1.16)		0.86(0.67–1.11)		0.87(0.68–1.12)		0.78(0.61–1.01)		0.81(0.63–1.05)
Sex	0.238		0.736		0.463		0.655		0.967		0.808	
Female		1		1		1		1		1		1
Male		0.81(0.56–1.15)		1.08(0.72–1.60)		1.16(0.78–1.75)		1.10(0.73–1.64)		0.99(0.67–1.47)		105(0.71–1.56)
ECOG PS	< 0.001		< 0.001		< 0.001		< 0.001		< 0.001		< 0.001	
0–1		1		1		1		1		1		1
2		3.86(2.71–5.50)		3.34(2.31–4.84)		3.41(2.34–4.97)		3.96(2.72–5.76)		3.77(2.60–5.45)		4.29(2.94–6.26)
Smoke history	< 0.001		< 0.001		0.016		0.005		0.008		< 0.001	
No		1		1		1		1		1		1
Yes		0.65(0.51–0.83)		0.62(0.48–0.81)		0.72(0.55–0.94)		0.69(0.53–0.90)		0.70(0.54–0.91)		0.62(0.48–0.81)
Stage	0.193		0.043		0.512		0.482		0.565		0.351	
IIIB		1		1		1		1		1		1
IIIC	0.167	1.77(0.79–3.99)	0.029	2.77(1.11–6.89)	0.708	1.18(0.49–2.85)	0.562	1.30(0.53–3.18)	0.565	1.29(0.54–3.10)	0.368	1.50(0.62–3.62)
IV	0.573	0.88(0.57–1.37)	0.868	0.96(0.61–1.52)	0.358	0.75(0.58–0.97)	0.432	0.83(0.53–1.31)	0.4	0.82(0.52–1.30)	0.333	0.80(0.51–1.26)
PD-L1 expression	0.032		0.413		0.448		0.246		0.439		0.624	
< 1%		1		1		1		1		1		1
≥ 1%		0.72(0.53–0.97)		0.88(0.64–1.22)		0.88(0.63–1.22)		0.83(0.60–1.14)		0.88(0.64–1.21)		0.92(0.67–1.27)
Treatment	0.02		0.002		0.032		0.006		0.044		0.017	
Monotherapy		1		1		1		1		1		1
Combination therapy		0.75(0.59–0.95)		0.67(0.5–0.87)		0.75(0.58–0.97)		0.69(0.53–0.90)		0.76(0.59–0.99)		0.72(0.55–0.94)
CEA	< 0.001		< 0.001									
3-Fold(> 15)		1		1								
Normal to 3-Fold (5–15)	0.246	0.85(0.64–1.12)	0.137	0.81(0.61–1.07)								
Normal (≤ 5.0)	< 0.001	0.38(0.28–0.52)	< 0.001	0.33(0.23–0.46)								
NSE	< 0.001				< 0.001							
3-Fold(> 48.9)		1				1						
Normal to 3-Fold (16.3–48.9)	< 0.001	0.37(0.24–0.58)			< 0.001	0.41(0.26–0.66)						
Normal (≤ 16.3)	< 0.001	0.24(0.15–0.39)			< 0.001	0.29(0.18–0.47)						
CYFRA21-1	0.011						0.004					
3-Fold(> 9.9)		1						1				
Normal to 3-Fold (3.3–9.9)	0.033	0.75(0.58–0.98)					0.006	0.69(0.53–0.90)				
Normal (≤ 3.3)	0.007	0.57(0.37–0.86)					0.003	0.53(0.35–0.80)				
CA19-9	< 0.001								< 0.001			
3-Fold(> 81)		1								1		
Normal to 3-Fold (27–81)	0.042	0.67(0.44–0.99)							0.063	0.68(0.45–1.02)		
Normal (≤ 27.0)	< 0.001	0.43(0.30–0.64)							< 0.001	0.45(0.31–0.67)		
CA125	< 0.001										< 0.001	
3-Fold(> 105)		1										1
Normal to 3-Fold (35–105)	0.026	0.70(0.51–0.96)									< 0.001	0.54(0.39–0.76)
Normal (≤ 35.0)	< 0.001	0.41(0.29–0.59)									< 0.001	0.31(0.21–0.44)
**Overall survival**												
Age	0.132		0.057		0.102		0.085		0.008		0.017	
Old (≥ 65)		1		1		1		1		1		1
Young (< 65)		0.81(0.62–1.07)		0.76(0.57–1.01)		0.79(0.59–1.05)		0.78(0.59–1.03)		0.68(0.51–0.90)		0.71(0.53–0.94)
Sex	0.673		0.728		0.527		0.351		0.87		0.675	
Female		1		1		1		1		1		1
Male		0.92(0.62–1.36)		1.08(0.69–1.69)		1.15(0.74–1.82)		1.24(0.79–1.94)		1.04(0.67–1.61)		1.11(0.71–1.72)
ECOG PS	< 0.001		< 0.001		< 0.001		< 0.001		< 0.001		< 0.001	
0–1		1		1		1		1		1		1
2		4.11(2.87–5.87)		3.61(2.45–5.33)		3.69(2.49–5.46)		4.43(2.99–6.56)		4.31(2.93–6.35)		4.76(3.20–7.08)
Smoke history	0.089		0.117		0.653		0.166		0.219		0.102	
No		1		1		1		1		1		1
Yes		0.79(0.60–1.04)		0.79(0.59–1.06)		1.45(0.55–3.80)		0.81(0.61–1.09)		0.83(0.62–1.12)		0.78(0.58–1.05)
Stage	0.169		0.034		0.45		0.674		0.657		0.275	
IIIB		1		1		1		1		1		1
IIIC	0.059	2.34(0.97–5.65)	0.007	4.01(1.17–10.94)	0.884	0.93(0.69–1.26)	0.261	1.75(0.66–4.64)	0.321	1.62(0.62–4.22)	0.902	1.71(0.65–4.48)
IV	0.682	1.13(0.64–1.98)	0.519	1.21(0.68–2.17)	0.922	1.03(0.57–1.85)	0.832	1.07(0.60–1.91)	0.869	1.06(0.59–1.88)	0.831	0.94(0.53–1.68)
PD-L1 expression	0.037		0.756		0.867		0.831		0.759		0.935	
< 1%		1		1		1		1		1		1
≥ 1%		0.71(0.51–0.98)		1.06(0.74–1.51)		1.10(0.77–1.58)		0.96(0.68–1.36)		1.05(0.74–1.51)		1.02(0.71–1.45)
Treatment	0.122		0.001		0.019		0.003		0.036		0.009	
Monotherapy		1		1		1		1		1		1
Combination therapy		0.81(0.61–1.06)		0.62(0.46–0.82)		0.70(0.52–0.94)		0.65(0.48–0.77)		0.73(0.55–0.98)		0.67(0.50–0.91)
CEA	< 0.001		< 0.001									
3-Fold(> 15)		1		1								
Normal to 3-Fold (5–15)	0.523	0.90(0.66–1.23)	0.309	0.85(0.62–1.16)								
Normal (≤ 5.0)	< 0.001	0.33(0.23–0.48)	< 0.001	0.28(0.18–0.42)								
NSE	< 0.001				< 0.001							
3-Fold(> 48.9)		1				1						
Normal to 3-Fold (16.3–48.9)	< 0.001	0.37(0.24–0.59)			< 0.001	0.42(0.26–0.68)						
Normal (≤ 16.3)	< 0.001	0.22(0.14–0.36)			< 0.001	0.27(0.16–0.46)						
CYFRA21-1	< 0.001						< 0.001					
3-Fold(> 9.9)		1						1				
Normal to 3-Fold (3.3–9.9)	0.012	0.69(0.52–0.92)					< 0.001	0.58(0.43–0.77)				
Normal (≤ 3.3)	< 0.001	0.38(0.23–0.64)					< 0.001	0.37(0.22–0.62)				
CA19-9	< 0.001								< 0.001			
3-Fold(> 81)		1								1		
Normal to 3-Fold (27–81)	0.004	0.54(0.35–0.82)							< 0.001	0.48(0.31–0.73)		
Normal (≤ 27.0)	< 0.001	0.33(0.22–0.49)							< 0.001	0.29(0.19–0.44)		
CA125	< 0.001										< 0.001	
3-Fold(> 105)		1										1
Normal to 3-Fold (35–105)	0.007	0.62(0.43–0.88)									< 0.001	0.47(0.33–0.68)
Normal (≤ 35.0)	< 0.001	0.38(0.25–0.56)									< 0.001	0.28(0.19–0.42)

*ECOG PS* Eastern cooperative oncology group performance status, *PD-L1* Programmed death ligand-1, *CEA* Carcinoembryonic antigen, *NSE* Neuron-specific enolase, *CYFRA21-1* Cytokeratin fragment 19, *CA19-9* Carbohydrate antigen 19–9, *CA125* Carbohydrate antigen 125

By multivariate analysis, CEA (Group A vs. Group C, HR [95% CI] = 0.33 [0.23–0.46], *P* < 0.001), NSE (Group A vs. Group B, HR [95% CI] = 0.41 [0.26–0.66], *P* < 0.001; Group A vs. Group C, HR [95% CI] = 0.29 [0.18–0.47], *P* < 0.001), CYFRA21-1 (Group A vs. Group B, HR [95% CI] = 0.69 [0.53–0.90], *P* = 0.006; Group A vs. Group C, HR [95% CI] = 0.53 [0.35–0.80], *P* = 0.003), CA19-9 (Group A vs. Group C, HR [95% CI] = 0.45 [0.31–0.67], *P* < 0.001), and CA125 (Group A vs. Group B, HR [95% CI] = 0.54 [0.39–0.76], *P* < 0.001; Group A vs. Group C, HR [95% CI] = 0.31 [0.21–0.44], *P* < 0.001) levels in patients with LUAD were associated with significantly different PFS among subgroups (Table 3). Similarly, CEA (Group A vs. Group C, HR [95% CI] = 0.28 [0.18–0.42], *P* < 0.001), NSE (Group A vs. Group B, HR [95% CI] = 0.42 [0.26–0.68], *P* < 0.001; Group A vs. Group C, HR [95% CI] = 0.27 [0.16–0.46], *P* < 0.001), CYFRA21-1 (Group A vs. Group B, HR [95% CI] = 0.58 [0.43–0.77],* P* < 0.001; Group A vs. Group C, HR [95% CI] = 0.37 [0.22–0.62], *P* < 0.001), CA19-9 (Group A vs. Group B, HR [95% CI] = 0.48 [0.31–0.73], *P* < 0.001; Group A vs. Group C, HR [95% CI] = 0.29 [0.19–0.44], *P* < 0.001), and CA125 (Group A vs. Group B, HR [95% CI] = 0.47 [0.33–0.68], *P* < 0.001; Group A vs. Group C, HR [95% CI] = 0.28 [0.19–0.42], *P* < 0.001) levels in patients with LUAD were associated with significantly different OS among subgroups (Table 3).

### Analysis of patients with lung squamous carcinoma

**Fig. 4:**
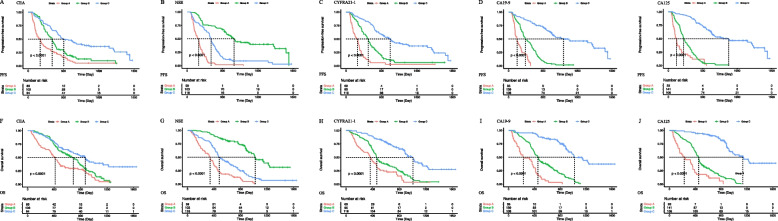
Kaplan–Meier curves of progression-free survival and overall survival in LUSC population. Kaplan–Meier curves are based on baseline CEA (A and F), NSE (B and G), CYFRA21-1 (C and H), CA19-9 (D and I), and CA125 (E and J) Group A: Baseline STM levels greater than three times the upper limit of the normal value. Group B: Baseline STM levels were higher than the upper limit of the normal value and less than three times the upper limit of the normal value. Group C: Baseline STM levels were lower than the upper limit of normal

Overall, the median PFS and OS periods of the 280 patients with LUSC were 336 days (95% CI, 292–385 days) and 623 days (95% CI, 505–759 days), respectively. By univariate analysis, CEA (Group A vs. Group C, HR [95% CI] = 0.49 [0.35–0.70], *P* < 0.001), NSE (Group A vs. Group B, HR [95% CI] = 0.74 [0.56–0.98], *P* < 0.001; Group A vs. Group C, HR [95% CI] = 0.21 [0.13–0.36], *P* < 0.001), CYFRA21-1 (Group A vs. Group B, HR [95% CI] = 0.74 [0.56–0.98], *P* = 0.035; Group A vs. Group C, HR [95% CI] = 0.44 [0.26–0.73], *P* = 0.001), and CA125 (Group A vs. Group B, HR [95% CI] = 0.44 [0.27–0.71], *P* = 0.01; Group A vs. Group C, HR [95% CI] = 0.45 [0.28–0.72], *P* < 0.001) levels in patients with LUSC were associated with significantly different PFS among subgroups (Table 4, Fig. 4). Similarly, CEA (Group A vs. Group C, HR [95% CI] = 0.39 [0.26–0.57], *P* < 0.001), NSE (Group A vs. Group B, HR [95% CI] = 0.38 [0.27–0.53], *P* < 0.001; Group A vs. Group C, HR [95% CI] = 0.23 [0.14–0.39], *P* < 0.001), CYFRA21-1 (Group A vs. Group B, HR [95% CI] = 0.59 [0.44–0.81], *P* < 0.001; Group A vs. Group C, HR [95% CI] = 0.38 [0.22–0.66], *P* < 0.001), and CA125 (Group A vs. Group B, HR [95% CI] = 0.45 [0.27–0.75], *P* = 0.002; Group A vs. Group C, HR [95% CI] = 0.37 [0.22–0.62], *P* < 0.001) levels in patients with LUSC were associated with significantly different OS among subgroups (Table 4, Fig. 4).

**Table 4:** Prognostic factors for progression-free survival and overall survival in patients with lung squamous cell carcinoma

Covariate	Univariate analysis		Multivariate analysis (CEA)		Multivariate analysis (NSE)		Multivariate analysis (CYFRA21-1)		Multivariate analysis (CA19-9)		Multivariate analysis (CA125)	
	*P*	HR (95% CI)	*P*	HR (95% CI)	*P*	HR (95% CI)	*P*	HR (95% CI)	*P*	HR (95% CI)	*P*	HR (95% CI)
**Progression-free survival**												
Age	0.625		0.8		0.336		0.782		0.642		0.682	
Old (≥ 65)		1		1		1		1		1		1
Young (< 65)		0.93(0.72–1.22)		1.04(0.77–1.39)		1.16(0.86–1.57)		1.04(0.78–1.40)		1.07(0.80–1.44)		1.06(0.79–1.43)
Sex	0.02		0.186		0.041		0.314		0.102		0.158	
Female		1		1		1		1		1		1
Male		1.52(1.06–2.17)		1.32(0.87–2.01)		1.55(1.02–2.35)		1.24(0.81–1.89)		1.41(0.93–2.14)		1.35(0.89–2.04)
ECOG PS	< 0.001		0.005		< 0.001		0.004		0.003		0.001	
0–1		1		1		1		1		1		1
2		2.30(1.52–3.48)		0.90(1.22–2.96)		2.19(1.40–3.43)		1.92(1.22–2.99)		1.96(1.26–3.07)		2.05(1.32–3.19)
Smoke history	0.006		0.372		0.119		0.108		0.147		0.179	
No		1		1		1		1		1		1
Yes		1.45(1.11–1.88)		1.17(0.83–1.63)		1.30(0.94–1.80)		1.31(0.94–1.81)		1.28(0.92–1.78)		1.25(0.90–1.77)
Stage	< 0.001		0.007		0.008		0.006		0.002		< 0.001	
IIIB		1				1				1		1
IIIC	< 0.001	3.20(1.71–5.99)	0.002	2.82(1.46–5.47)	0.001	3.00(1.55–5.82)	0.005	2.61(1.33–5.10)	0.002	2.87(1.48–5.58)	< 0.001	3.29(1.69–6.38)
IV	0.005	1.70(1.17–2.45)	0.017	1.59(1.08–2.34)	0.011	1.64(1.12–2.40)	0.006	1.70(1.16–2.48)	0.006	1.70(1.16–2.50)	0.005	1.72(1.18–2.52)
PD-L1 expression	0.774		0.658		0.485		0.383		0.665		0.846	
< 1%		1		1		1		1		1		1
≥ 1%		0.97(1.05–1.30)		1.08(0.77–1.51)		1.13(0.80–1.59)		1.16(0.83–1.64)		1.08(0.77–1.51)		0.97(0.69–1.36)
Treatment	0.553		0.628		363		0.489		0.708		0.562	
Monotherapy		1		1		1		1		1		1
Combination therapy		1.09(0.82–1.44)		0.93(0.68–1.27)		0.87(0.63–1.18)		0.90(0.65–1.23)		0.94(0.69–1.29)		0.91(0.67–1.24)
CEA	< 0.001		0.041									
3-Fold(> 15)		1		1								
Normal to 3-Fold (5–15)	0.058	0.68(0.46–1.01)	0.233	0.78(0.51–1.18)								
Normal (≤ 5.0)	< 0.001	0.49(0.35–0.70)	0.023	0.64(0.44–0.94)								
NSE	< 0.001				< 0.001							
3-Fold(> 48.9)		1				1						
Normal to 3-Fold (16.3–48.9)	< 0.001	0.36(0.22–0.58)			< 0.001	0.31(0.19–0.52)						
Normal (≤ 16.3)	< 0.001	0.21(0.13–0.36)			< 0.001	0.18(0.11–0.31)						
CYFRA21-1	0.013						0.036					
3-Fold(> 9.9)		1						1				
Normal to 3-Fold (3.3–9.9)	0.035	0.74(0.56–0.98)					0.303	0.86(0.64–1.15)				
Normal (≤ 3.3)	0.001	0.44(0.26–0.73)					0.013	0.51(0.30–0.87)				
CA19-9	0.754								0.857			
3-Fold(> 81)		1								1		
Normal to 3-Fold (27–81)	0.911	1.03(0.57–1.89)							0.715	1.12(0.61–2.07)		
Normal (≤ 27.0)	0.553	0.84(0.48–1.49)							0.952	1.02(0.57–1.83)		
CA125	< 0.001										< 0.001	
3-Fold(> 105)		1										1
Normal to 3-Fold (35–105)	< 0.001	0.44(0.27–0.71)									< 0.001	0.41(0.25–0.69)
Normal (≤ 35.0)	< 0.001	0.45(0.28–0.72)									0.004	0.48(0.29–0.79)
**Overall survival**												
Age	0.006		0.057		0.107		0.054		0.055		0.062	
Old (≥ 65)		1		1		1		1		1		1
Young (< 65)		0.67(0.50–0.89)		0.73(0.53–1.01)		0.76(0.55–1.06)		0.73(0.52–1.01)		0.73(0.53–1.01)		0.73(0.53–1.02)
Sex	0.003		0.068		0.041		0.147		0.033		0.053	
Female		1		1		1		1		1		1
Male		1.88(1.24–2.85)		1.56(0.97–2.52)		1.65(1.02–2.66)		1.43(0.88–2.32)		1.56(0.95–2.56)		1.60(0.99–2.58)
ECOG PS	< 0.001		< 0.001		< 0.001		< 0.001		< 0.001		< 0.001	
0–1		1		1		1		1		1		1
2		2.30(1.50–3.55)		2.36(1.49–3.73)		2.71(1.70–4.34)		2.24(1.40–3.60)		2.34(1.46–3.73)		2.46(1.55–3.90)
Smoke history	< 0.001		0.542		0.126		0.104		0.271		0.244	
No		1		1		1		1		1		1
Yes		1.77(1.33–2.36)		1.12(0.78–1.60)		1.33(0.92–1.91)		1.34(0.94–1.92)		1.22(0.85–1.76)		1.23(0.86–1.77)
Stage	0.062		0.215		0.313		0.268		0.172		0.115	
IIIB		1				1				1		1
IIIC	0.078	1.83(0.93–3.57)	0.136	1.70(0.85–3.39)	0.338	1.42(0.70–2.88)	0.333	1.42(0.69–2.90)	0.151	1.68(0.83–3.40)	0.086	1.85(0.92–3.74)
IV	0.14	1.35(0.91–1.99)	0.291	1.24(0.83–1.86)	0.228	1.28(0.86–1.92)	0.217	1.29(0.86–1.93)	0.191	1.31(0.87–1.96)	0.173	1.32(0.88–1.97)
PD-L1 expression	0.023		0.344		0.193		0.558		0.196		0.079	
< 1%		1		1		1		1		1		1
≥ 1%		0.68(0.49–0.95)		0.84(0.59–1.20)		0.79(0.55–1.13)		0.97(0.70–1.35)		0.79(0.55–1.13)		0.72(0.50–1.04)
Treatment	0.948		0.847		0.878		0.879		0.67		0.768	
Monotherapy		1		1		1		1		1		1
Combination therapy		0.99(0.73–1.35)		1.03(0.74–1.43)		1.03(0.74–1.43)		0.97(0.70–1.35)		1.07(0.77–1.50)		1.05(0.76–1.46)
CEA	< 0.001		< 0.001									
3-Fold(> 15)		1		1								
Normal to 3-Fold (5–15)	0.106	0.71(0.46–1.08)	0.146	0.73(0.47–1.12)								
Normal (≤ 5.0)	< 0.001	0.39(0.26–0.57)	< 0.001	0.48(0.32–0.73)								
NSE	< 0.001				< 0.001							
3-Fold(> 48.9)		1				1						
Normal to 3-Fold (16.3–48.9)	< 0.001	0.35(0.21–0.57)			< 0.001	0.33(0.20–0.56)						
Normal (≤ 16.3)	< 0.001	0.23(0.14–0.39)			< 0.001	0.22(0.13–0.37)						
CYFRA21-1	< 0.001						0.015					
3-Fold(> 9.9)		1						1				
Normal to 3-Fold (3.3–9.9)	< 0.001	0.59(0.44–0.81)					0.028	0.70(0.51–0.96)				
Normal (≤ 3.3)	< 0.001	0.38(0.22–0.66)					0.005	0.44(0.24–0.78)				
CA19-9	0.616								0.473			
3-Fold(> 81)		1								1		
Normal to 3-Fold (27–81)	0.84	0.94(0.49–1.80)							0.688	0.87(0.44–1.71)		
Normal (≤ 27.0)	0.238	0.69(0.37–1.28)							0.353	0.74(0.39–1.40)		
CA125	0.002										< 0.001	
3-Fold(> 105)		1										1
Normal to 3-Fold (35–105)	0.002	0.45(0.27–0.75)									< 0.001	0.38(0.22–0.64)
Normal (≤ 35.0)	< 0.001	0.37(0.22–0.62)									< 0.001	0.36(0.22–0.61)

*ECOG PS* Eastern cooperative oncology group performance status, *PD-L1* Programmed death ligand-1, *CEA* Carcinoembryonic antigen, *NSE* Neuron-specific enolase, *CYFRA21-1* Cytokeratin fragment 19, *CA19-9* Carbohydrate antigen 19–9, *CA125* Carbohydrate antigen 125

By multivariate analysis, CEA (Group A vs. Group C, HR [95% CI] = 0.64 [0.44–0.94], *P* = 0.023), NSE (Group A vs. Group B, HR [95% CI] = 0.31 [0.19–0.52], *P* < 0.001; Group A vs. Group C, HR [95% CI] = 0.18 [0.11–0.31], *P* < 0.001), CYFRA21-1 (Group A vs. Group C, HR [95% CI] = 0.51 [0.30–0.87], *P* = 0.013), and CA125 (Group A vs. Group B, HR [95% CI] = 0.41 [0.25–0.69], *P* < 0.001; Group A vs. Group C, HR [95% CI] = 0.48 [0.29–0.79], *P* = 0.004) levels in patients with LUSC were associated with significantly different PFS among subgroups (Table 4). Similarly, CEA (Group A vs. Group C, HR [95% CI] = 0.48 [0.32–0.73], *P* < 0.001), NSE (Group A vs. Group B, HR [95% CI] = 0.33 [0.20–0.56], *P* < 0.001; Group A vs. Group C, HR [95% CI] = 0.22 [0.13–0.37], *P* < 0.001), CYFRA21-1 (Group A vs. Group B, HR [95% CI] = 0.70 [0.51–0.96],* P* = 0.028; Group A vs. Group C, HR [95% CI] = 0.44 [0.24–0.78], *P* = 0.005), and CA125 (Group A vs. Group B, HR [95% CI] = 0.38 [0.22–0.64], *P* < 0.001; Group A vs. Group C, HR [95% CI] = 0.36 [0.22–0.61], *P* < 0.001) levels in patients with LUSC were associated with significantly different OS among subgroups (Table 4).

### Analysis of patients with non-small cell lung cancer who received monotherapy

By univariate analysis, NSE (Group A vs. Group B, HR [95% CI] = 0.44 [0.26–0.74], *P* = 0.002; Group A vs. Group C, HR [95% CI] = 0.16 [0.09–0.29], *P* < 0.001), CA19-9 (Group A vs. Group C, HR [95% CI] = 0.44 [0.32–0.97], *P* = 0.038), and CA125 (Group A vs. Group C, HR [95% CI] = 0.65[0.42–0.99], *P* = 0.045) levels were associated with significantly different PFS among subgroups (Table S3). Similarly, NSE (Group A vs. Group B, HR [95% CI] = 0.38 [0.22–0.63], *P* < 0.001; Group A vs. Group C, HR [95% CI] = 0.25 [0.15–0.43], *P* < 0.001), CYFRA21-1 (Group A vs. Group B, HR [95% CI] = 0.71 [0.51–0.98], *P* = 0.039; Group A vs. Group C, HR [95% CI] = 0.49 [0.24–0.76], *P* = 0.004), CA19-9 (Group A vs. Group C, HR [95% CI] = 0.56 [0.25–0.77], *P* = 0.004), and CA125 (Group A vs. Group C, HR [95% CI] = 0.61 [0.38–0.98], *P* = 0.039) levels were associated with significantly different OS among subgroups (Table S3).

By multivariate analysis, CEA (Group A vs. Group C, HR [95% CI] = 0.60[0.42–0.86], *P* = 0.003), NSE (Group A vs. Group B, HR [95% CI] = 0.39[0.23–0.67], *P* < 0.001; Group A vs. Group C, HR [95% CI] = 0.23[0.13–0.40], *P* < 0.001), CA19-9 (Group A vs. Group C, HR [95% CI] = 0.48[0.27–0.86], *P* = 0.013), and CA125 (Group A vs. Group B, HR [95% CI] = 0.49 [0.37–0.64], *P* < 0.001; Group A vs. Group C, HR [95% CI] = 0.43 [0.27–0.67], *P* < 0.001) levels were associated with significantly different PFS among subgroups (Table S3). Similarly, by multivariate analysis, CEA (Group A vs. Group C, HR [95% CI] = 0.64[0.43–0.95], *P* = 0.026), NSE (Group A vs. Group B, HR [95% CI] = 0.31 [0.18–0.53], *P* < 0.001; Group A vs. Group C, HR [95% CI] = 0.14[0.08–0.25), *P* < 0.001), CYFRA21-1 ( Group A vs. Group C, HR [95% CI] = 0.49 [0.28–0.89], *P* = 0.018), CA19-9 (Group A vs. Group C, HR [95% CI] = 0.33[0.18–0.60], *P* < 0.001), and CA125 ( Group A vs. Group C, HR [95% CI] = 0.41 [0.25–0.68], *P* < 0.001) levels were associated with significantly different OS among subgroups (Table S3).

### Analysis of patients with non-small cell lung cancer who received combination therapy

By univariate analysis, CEA (Group A vs. Group C, HR [95% CI] = 0.40 [0.30–0.52], *P* < 0.001), NSE (Group A vs. Group B, HR [95% CI] = 0.37 [0.24–0.56], *P* < 0.001; Group A vs. Group C, HR [95% CI] = 0.27 [0.17–0.41], *P* < 0.001), CYFRA21-1 (Group A vs. Group B, HR [95% CI] = 0.65 [0.52–0.82], *P* < 0.001; Group A vs. Group C, HR [95% CI] = 0.40 [0.27–0.59], *P* < 0.001), CA19-9 (Group A vs. Group C, HR [95% CI] = 0.56[0.39–0.82], *P* = 0.003), and CA125 (Group A vs. Group B, HR [95% CI] = 0.38 [0.27–0.54], *P* < 0.001; Group A vs. Group C, HR [95% CI] = 0.32 [0.23–0.46], *P* < 0.001) levels were associated with significantly different PFS among subgroups (Table S4). Similarly, NSE (Group A vs. Group B, HR [95% CI] = 0.38 [0.22–0.63], *P* < 0.001; Group A vs. Group C, HR [95% CI] = 0.25 [0.15–0.43], *P* < 0.001), CYFRA21-1 (Group A vs. Group B, HR [95% CI] = 0.61[0.47–0.79], *P* < 0.001; Group A vs. Group C, HR [95% CI] = 0.35 [0.22–0.55], *P* < 0.001), CA19-9 (Group A vs. Group B, HR [95% CI] = 0.59 [0.38–0.91], *P* = 0.018; Group A vs. Group C, HR [95% CI] = 0.46 [0.31–0.69], *P* < 0.001), and CA125 (Group A vs. Group B, HR [95% CI] = 0.33 [0.23–0.48], *P* < 0.001; Group A vs. Group C, HR [95% CI] = 0.29 [0.20–0.43], *P* < 0.001) levels were associated with significantly different OS among subgroups (Table S4).

By multivariate analysis, CEA (Group A vs. Group C, HR [95% CI] = 0.36(0.27–0.48), *P* < 0.001), NSE (Group A vs. Group B, HR [95% CI] = 0.35 [0.23–0.54], *P* < 0.001; Group A vs. Group C, HR [95% CI] = 0.25 [0.16–0.39], *P* < 0.001), CYFRA21-1 (Group A vs. Group B, HR [95% CI] = 0.69 [0.54–0.88], *P* = 0.002; Group A vs. Group C, HR [95% CI] = 0.46 [0.31–0.68], *P* < 0.001), CA19-9 ( Group A vs. Group C, HR [95% CI] = 0.58 [0.39–0.85], *P* = 0.005), and CA125 (Group A vs. Group B, HR [95% CI] = 0.36 [0.25–0.51], *P* < 0.001; Group A vs. Group C, HR [95% CI] = 0.32 [0.22–0.46], *P* < 0.001) levels were associated with significantly different PFS among subgroups (Table S4). Similarly, by multivariate analysis, CEA (Group A vs. Group C, HR [95% CI] = 0.32 [0.23–0.44], *P* < 0.001), NSE (Group A vs. Group B, HR [95% CI] = 0.47 [0.30–0.74], *P* = 0.001; Group A vs. Group C, HR [95% CI] = 0.36[0.23–0.58], *P* < 0.001), CYFRA21-1 (Group A vs. Group B, HR [95% CI] = 0.66[0.51–0.86], *P* = 0.002; Group A vs. Group C, HR [95% CI] = 0.41[0.26–0.66], *P* < 0.001), CA19-9 (Group A vs. Group B, HR [95% CI] = 0.58 [0.39–0.85], *P* = 0.005; Group A vs. Group C, HR [95% CI] = 0.45 [0.30–0.68], *P* < 0.001), and CA125 (Group A vs. Group B, HR [95% CI] = 0.63 [0.20–0.44], *P* < 0.001; Group A vs. Group C, HR [95% CI] = 0.28 [0.19–0.42), *P* < 0.001) levels were associated with significantly different OS among subgroups (Table S4).

### Association between STM levels and programmed apoptosis ligand 1 expression

All patients underwent PD-L1 testing, and 154 of them were negative for PD-L1 expression (Table 1). In patients with LUAD, only CA19-9 concentration and PD-L1 expression were statistically different (Table 5). In patients with LUSC, there was no statistical difference between STM concentrations and PD-L1 expression (Table 5).

**Table 5:** The association with serum tumor markers and PD-L1 expression levels in patients

Serum tumor markers	**LUAD** (*N* = 390)			**LUSC** (*N* = 280)		
	PD-L1( +)	PD-L1 (-)	*P* value	PD-L1( +)	PD-L1 (-)	*P* value
**CEA (ng/ml)**			0.161			0.242
Normal (≤ 5.0)	100	17		78	30	
High (> 5.0)	215	58		136	36	
**NSE (ng/ml)**			0.675			1
Normal (≤ 16.3)	137	30		89	27	
High (> 16.3)	178	45		125	39	
**CYFRA21-1 (ng/ml)**			0.795			0.104
Normal (≤ 3.3)	44	9		27	3	
High (> 3.3)	271	66		187	63	
**CA19-9 (ng/ml)**			0.043			0.956
Normal (≤ 27.0)	194	36		143	45	
High (> 27.0)	121	39		71	21	
**CA125 (ng/ml)**						
Normal (≤ 35.0)	114	23	0.444	109	30	0.524
High (> 35.0)	201	52		105	36	

*LUAD* Lung adenocarcinoma, *LUSC* Lung squamous cell carcinoma, *PD-L1* Programmed death ligand-1, *CEA* Carcinoembryonic antigen, *NSE* Neuron-specific enolase, *CYFRA21-1* Cytokeratin fragment 19, *CA19-9* Carbohydrate antigen 19–9, *CA125* Carbohydrate antigen 125

### Correlation between STM levels and tumor response

In patients with LUAD, the ORRs of the STM (CEA, NSE, CA19-9, and CA125) groups were statistically different (*P* < 0.001, *P* < 0.001, *P* = 0.014, and *P* = 0.002, respectively) (Table 6). In addition, the DCRs of the STMs = (CEA, NSE, CYFRA 21–1, and CA19-9) groups were statistically different (*P* < 0.001, *P* < 0.001, *P* = 0.007, and *P* = 0.001, respectively) (Table 6).

**Table 6:** The association with serum tumor markers and ORRs and DCRs

Serum tumor markers	**LUAD** (*N* = 390)						**LUSC** (*N* = 280)					
**CEA group**	ORR	Non-ORR	*P* value	DCR	Non-DCR	*P* value	ORR	Non-ORR	*P* value	DCR	Non-DCR	*P* value
A^*^	52	119	< 0.001	141	30	< 0.001	14	33	0.269	39	8	0.006
B	51	51		95	7		23	44		56	11	
C	70	47		112	5		69	97		157	9	
**NSE group**												
A	2	22	< 0.001	11	13	< 0.001	7	12	0.873	14	5	0.005
B	88	111		177	22		53	92		127	18	
C	83	84		160	7		46	70		111	5	
**CYFRA21-1 group**												
A	50	80	0.212	108	22	0.007	49	87	0.553	117	19	0.107
B	96	111		188	19		43	71		107	7	
C	27	26		52	1		14	16		28	2	
**CA19-9 group**												
A	13	26	0.014	31	8	0.001	7	10	0.206	17	0	0.013
B	44	77		101	20		22	53		61	14	
C	116	114		216	14		77	111		174	14	
**CA125 group**												
A	20	43	0.002	53	10	0.102	4	18	0.016	16	6	0.025
B	77	113		167	23		39	80		110	9	
C	76	61		128	9		63	76		126	13	

*LUAD* Lung adenocarcinoma, *LUSC* Lung squamous cell carcinoma, *CEA* Carcinoembryonic antigen, *NSE* Neuron-specific enolase, *CYFRA21-1* Cytokeratin fragment 19, *CA19-9* Carbohydrate antigen 19–9, *CA125* Carbohydrate antigen 125, *ORR* Objective response rate, *DCR* Disease control rate

^*^Group A: Level of baseline serum tumor markers greater than 3 times the upper limit of normal value

Group B: Level of baseline serum tumor markers was higher than the upper limit of normal value and lower than 3 times the upper limit of normal value

Group C: Level of baseline serum tumor markers were lower than the upper limit of normal

In patients with LUSC, the ORRs of the STM (CA125) groups were statistically different (*P* = 0.016), and the DCRs of the STM (CEA, NSE, CA19-9, and CA125) groups were statistically different (*P* = 0.006, *P* = 0.005, *P* = 0.013, and *P* = 0.025, respectively) (Table 6).

## Discussion

Several large-scale clinical studies have confirmed the efficacy of first-line immunotherapy in improving survival in patients with advanced NSCLC [3–6], but screening the potential patients who can benefit from immunotherapy before treatment remains unclear [10]. PD-L1, the most commonly used biomarker to predict the efficacy of immunotherapy, has limitations [8, 9, 18].

As routinely measured clinical biomarkers, STMs have been used on a large scale in the diagnosis of malignancies and in the prediction of efficacy [19–23]. To date, several studies have reported an association between baseline or dynamic STMs and the efficacy of immunotherapy [24–27]. However, all these studies have limitations. First, previous studies did not specifically focus on patients receiving first-line immunotherapy and included a small number of patients, which may have led to potential bias [24–27]. To the best of our knowledge, to date, our study is the largest study to investigate the association between baseline STM levels and first-line immunotherapy efficacy. Second, previous studies simply dichotomized the concentration of STMs, making it difficult to distinguish patients who can benefit from immunotherapy more precisely [24–26]. In our study, we used the upper limit of the normal value of STMs and threefold the upper limit of the normal value of STMs as cut-off values to divide the STMs into three groups. This classification was used to confirm whether STM concentrations were associated with immunotherapy efficacy.

CEA, the classical and most widely used STM [28], had superior PFS and OS at baseline CEA levels, lower than the upper limit of normal in patients with LUAD and LUSC in our cohort. However, higher baseline CEA levels are not associated with worse prognosis. Therefore, in clinical practice, we do not predict patient survival based on serum CEA concentrations if they are above the upper limit of the normal value.

NSE is commonly used in small cell lung cancers [29]. Although serum NSE level is also elevated in patients with NSCLC, dynamic changes in serum NSE levels are controversial in predicting the efficacy of immunotherapy. Bello et al. reported that dynamic monitoring of NSE levels could not predict survival in patients with NSCLC treated with immunotherapy [26]. However, Chen et al. reported that dynamic monitoring of NSE levels could predict survival in patients with NSCLC treated with immunotherapy [24]. Both studies included a small number of patients, did not differentiate pathological subtypes, and included less than 50% of the patients who received first-line immunotherapy [24, 26]. Therefore, there may have been potential bias. Baseline NSE levels predict survival in patients with advanced NSCLC treated with targeted therapy. However, no studies have confirmed an association between baseline NSE levels and immunotherapy efficacy. In our study, baseline NSE levels predicted first-line immunotherapy efficacy in patients with LUAD and LUSC; the higher the baseline NSE levels, the inferior the PFS and OS periods. We found that the serum baseline NSE levels and PD-L1 expression were independent of each other. Therefore, these levels can be used together with PD-L1 expression as an indicator of efficacy and survival prediction in future clinical practice.

CYFRA21-1 has also been widely used as a tumor marker for NSCLC in recent years [19, 20]. We found that, similar to NSE, it predicted PFS and OS with first-line immunotherapy, except that it did not lead to inferior PFS with higher concentrations of CYFRA21-1 at baseline in patients with LUAD.

CA19-9 is mainly used in digestive tract tumors but is also elevated in NSCLC [23]. We found that in patients with LUAD, baseline CA19-9 level remained a valid predictor, and a higher CA19-9 level was associated with inferior OS. However, in patients with LUSC, CA19-9 level was not an independent prognostic factor.

CA125 is a classic tumor marker in ovarian cancer [21], but more than half of the patients with advanced NSCLC have CA125 levels higher than the upper limit of the normal value. In our study, CA125, similar to NSE, can effectively predict patients who can benefit from immunotherapy, and the higher the baseline CA125 level, the inferior the PFS and OS periods.

STMs can also serve as powerful complements to the treatment response in patients with LUAD. The baseline levels of CEA, NSE, and CA19-9 were statistically different from those of ORR and DCR. However, this phenomenon is not evident in patients with LUSC, suggesting the complexity of immunotherapy treatment response and efficacy prediction.

Our study has some limitations. First, this retrospective study had some selection bias. Second, there was no dynamic monitoring of STMs, and it was difficult to comprehensively evaluate the value of STMs in immunotherapy. Third, lung cancer is a highly heterogeneous malignant tumor, and whether the increase in NSE was due to a combination of small cells was not determined in this study.

To the best of our knowledge, this is the largest multicenter retrospective study to investigate the efficacy of baseline STMs in first-line immunotherapy. We further subdivided the patients into three groups based on STM levels and further subdivided the patients who could benefit from immunotherapy. It was also confirmed that the level of certain STMs at baseline affects immunotherapy efficacy rather than simply dichotomizing STMs based on the upper limit of the normal value. In addition, we found that PD-L1 expression levels were independent of most STM levels. Therefore, STMs can be used as effective prognostic factors in addition to PD-L1 in subsequent clinical applications. Finally, STMs routinely measured in clinics is an easy and effective tool to predict the efficacy of immunotherapy.

## Conclusion

In patients with LUAD, STMs predict PFS and OS with first-line immunotherapy; higher serum NSE, CYFRA21-1, and CA125 levels are associated with inferior PFS. In addition, higher serum NSE, CYFRA21-1, CA19-9, and CA125 levels are associated with inferior OS. In patients with LUSC, serum CEA, NSE, CYFRA21-1, and CA125 levels predict PFS and OS with first-line immunotherapy, and higher serum NSE, CYFRA21-1, and CA125 levels are associated with inferior PFS and OS. These findings need to be validated in large prospective studies.

### Supplementary Information

**Additional file 1.**

## Data Availability

The datasets used or analyzed during the current study are available from the corresponding author on reasonable request.
