# Supplementary material for: Baseline serum tumor markers predict the survival of patients with advanced non-small cell lung cancer receiving first-line immunotherapy: a multicenter retrospective study
Source: BMC Cancer. 2023 Aug 30;23:812. doi: 10.1186/s12885-023-11312-4 (PMC10466830; doi:10.1186/s12885-023-11312-4)
Supplement: Supplementary file 1 — Additional file 1. [file 12885_2023_11312_MOESM1_ESM.docx]

| **Table S1.** Characteristics of patients with LUAD and LUSC. | | |
| --- | --- | --- |
| Characteristics | Patients with LUAD (N = 390) | Patients with LUSC (N = 280) |
| **Age** (Mean ± SD) | 61.33 ± 10.14 | 60.11 ± 11.19 |
| **Sex** |  |  |
| Male | 345 | 226 |
| Female | 45 | 54 |
| **Clinical stage** |  |  |
| IIIB | 30 | 52 |
| IIIC | 9 | 16 |
| IV | 351 | 212 |
| **Smoking history** |  |  |
| Never smoker | 164 | 144 |
| Smoker or ex-smoker | 226 | 136 |
| **PD-L1 expression** |  |  |
| < 1% | 75 | 66 |
| 1%-49% | 146 | 134 |
| ≥ 50% | 169 | 80 |
| **Treatment type** |  |  |
| Monotherapy | 176 | 88 |
| Combination therapy | 214 | 192 |
| **ECOG PS** |  |  |
| 0–1 | 349 | 256 |
| 2 | 41 | 24 |
| **Radiation history** |  |  |
| Yes | 263 | 159 |
| No | 127 | 121 |
| **Metastasis sites** |  |  |
| Liver | 27 | 30 |
| Lung | 101 | 70 |
| Brain | 93 | 26 |
| Bone | 127 | 72 |
| Adrenal | 94 | 16 |
| **Drug** |  |  |
| Pembrolizumab | 255 | 162 |
| Nivolumab | 48 | 58 |
| Atezolizumab | 1 | 4 |
| Sintilimab | 56 | 44 |
| Camrelizumab | 23 | 8 |
| Tislelizumab | 7 | 4 |
| **CEA (ng/ml)** |  |  |
| Mean ± SD | 90.56 ± 200.75 | 19.36 ± 75.74 |
| Normal (≤ 5.0) | 117 | 166 |
| High (> 5.0) | 273 | 114 |
| **NSE (ng/ml)** |  |  |
| Mean ± SD | 24.00 ± 16.40 | 24.55 ± 18.83 |
| Normal (≤ 16.3) | 167 | 116 |
| High (> 16.3) | 223 | 164 |
| **CYFRA21-1 (ng/ml)** |  |  |
| Mean ± SD | 13.53 ± 24.79 | 20.24 ± 31.79 |
| Normal (≤ 3.3) | 53 | 30 |
| High (> 3.3) | 337 | 250 |
| **CA19-9 (ng/ml)** |  |  |
| Mean ± SD | 55.83 ± 132.40 | 35.28 ± 64.59 |
| Normal (≤ 27.0) | 230 | 188 |
| High (> 27.0) | 160 | 92 |
| **CA125 (ng/ml)** |  |  |
| Mean ± SD | 87.49 ± 141.94 | 49.37 ± 58.42 |
| Normal (≤ 35.0) | 137 | 139 |
| High (> 35.0) | 235 | 141 |

| **Table S2.** Dynamic changes of STMs before and after treatment. | |
| --- | --- |
| Dynamic changes of STMs | Patients (N = 716) |
| **CEA** |  |
| ≥20% (Increased) | 190 |
| -20% to 20% | 228 |
| ≥20% (Decreased) | 298 |
| **NSE** |  |
| ≥20% (Increased) | 133 |
| -20% to 20% | 203 |
| ≥20% (Decreased) | 303 |
| **CYFRA21-1** |  |
| ≥20% (Increased) | 165 |
| -20% to 20% | 218 |
| ≥20% (Decreased) | 333 |
| **CA19-9** |  |
| ≥20% (Increased) | 87 |
| -20% to 20% | 298 |
| ≥20% (Decreased) | 331 |
| **CA125** |  |
| ≥20% (Increased) | 80 |
| -20% to 20% | 294 |
| ≥20% (Decreased) | 342 |

| **Table S3.** Prognostic factors for progression-free survival and overall survival in patients with advanced non-small cell lung cancer who received monotherapy. | | | | | | | | | | | | |
| --- | --- | --- | --- | --- | --- | --- | --- | --- | --- | --- | --- | --- |
| Covariate | Univariate analysis | | Multivariate analysis (CEA) | | Multivariate analysis (NSE) | | Multivariate analysis (CYFRA21-1) | | Multivariate analysis (CA19-9) | | Multivariate analysis (CA125) | |
|  | *P* | HR (95% CI) | *P* | HR (95% CI) | *P* | HR (95% CI) | *P* | HR (95% CI) | *P* | HR (95% CI) | *P* | HR (95% CI) |
| **Progression-free survival** |  |  |  |  |  |  |  |  |  |  |  |  |
| Age | 0.954 |  | 0.129 |  | 0.201 |  | 0.143 |  | 0.089 |  | 0.127 |  |
| Old ( ≥ 65) |  | 1 |  | 1 |  | 1 |  | 1 |  | 1 |  | 1 |
| Young (< 65) |  | 0.99(0.75-1.31) |  | 0.79(0.58-1.07) |  | 0.82(0.61-1.11) |  | 0.79(0.58-1.08) |  | 0.76(0.56-1.04) |  | 0.79(0.58-1.07) |
| Sex | 0.796 |  | 0.528 |  | 0.173 |  | 0.453 |  | 0.185 |  | 0.256 |  |
| Female |  | 1 |  | 1 |  | 1 |  | 1 |  | 1 |  | 1 |
| Male |  | 1.05(0.71-1.55) |  | 1.16(0.73-1.83) |  | 1.38(0.87-2.21) |  | 1.19(0.75-1.90) |  | 1.37(0.86-2.17) |  | 1.30(0.83-2.04) |
| ECOG PS | < 0.001 |  | < 0.001 |  | < 0.001 |  | < 0.001 |  | < 0.001 |  | < 0.001 |  |
| 0-1 |  | 1 |  | 1 |  | 1 |  | 1 |  | 1 |  | 1 |
| 2 |  | 3.51(2.44-5.04) |  | 3.31(2.26-4.86) |  | 3.55(2.43-5.20) |  | 3.31(2.26-4.87) |  | 3.41(2.31-5.03) |  | 3.90(2.63-5.78) |
| Smoke history | 0.298 |  | 0.084 |  | 0.117 |  | 0.189 |  | 0.081 |  | 0.032 |  |
| No |  | 1 |  | 1 |  | 1 |  | 1 |  | 1 |  | 1 |
| Yes |  | 0.86(0.65-1.14) |  | 0.75(0.54-1.04) |  | 0.77(0.56-1.07) |  | 0.81(0.59-1.11) |  | 0.75(0.54-1.04) |  | 0.70( 0.50-0.97) |
| Histological type | 0.001 |  | < 0.001 |  | 0.036 |  | 0.033 |  | 0.021 |  | 0.013 |  |
| LUAD |  | 1 |  | 1 |  | 1 |  | 1 |  | 1 |  | 1 |
| LUSC | 0.001 | 1.65(1.22-2.23) | < 0.001 | 1.76(1.26-2.47) | 0.015 | 1.49(1.08-2.08) | 0.014 | 1.51(1.09-2.11) | 0.004 | 1.61(1.16-2.24) | 0.004 | 1.62(1.17-2.25) |
| Other_NSCLC | 0.797 | 0.92(0.51-1.68) | 0.669 | 0.87(0.47-1.62) | 0.741 | 0.90(0.49-1.67) | 0.704 | 0.89(0.47-1.65) | 0.558 | 0.83(0.44-1.55) | 0.567 | 0.83(0.44-1.56) |
| Stage | 0.714 |  | 0.622 |  | 0.811 |  | 0.587 |  | 0.654 |  | 0.438 |  |
| IIIB |  | 1 |  | 1 |  | 1 |  | 1 |  | 1 |  | 1 |
| IIIC | 0.832 | 1.10(0.44-2.75) | 0.786 | 0.88(0.35-2.23) | 0.83 | 0.90(0.35-2.32) | 0.628 | 0.79(0.31-2.04) | 0.722 | 0.85(0.33-2.14) | 0.639 | 0.80(0.32-2.03) |
| IV | 0.449 | 0.79(0.43-1.45) | 0.489 | 0.80(0.43-1.51) | 0.709 | 0.88(1.13-1.69) | 0.494 | 0.80(0.42-1.53) | 0.548 | 0.82(0.44-1.55) | 0.331 | 0.73(0.39-1.37) |
| PD-L1 expression | 0.075 |  | 0.085 |  | 0.156 |  | 0.192 |  | 0.321 |  | 0.202 |  |
| < 1% |  | 1 |  | 1 |  | 1 |  | 1 |  | 1 |  | 1 |
| ≥ 1% |  | 0.67(0.44-1.04) |  | 0.67(0.43-1.51) |  | 0.72(0.46-1.13) |  | 0.74(0.46-1.17) |  | 0.79(0.50-1.26) |  | 0.74(0.47-1.17) |
| CEA | 0.435 |  | 0.011 |  |  |  |  |  |  |  |  |  |
| 3-Fold(＞15) |  | 1 |  | 1 |  |  |  |  |  |  |  |  |
| Normal to 3-Fold (5-15) | 0.543 | 0.89(0.63-1.28) | 0.198 | 0.78(0.54-1.14) |  |  |  |  |  |  |  |  |
| Normal (≤ 5.0) | 0.09 | 0.75(0.54-1.05) | 0.005 | 0.60(0.42-0.86) |  |  |  |  |  |  |  |  |
| NSE | < 0.001 |  |  |  | < 0.001 |  |  |  |  |  |  |  |
| 3-Fold(＞48.9) |  | 1 |  |  |  | 1 |  |  |  |  |  |  |
| Normal to 3-Fold (16.3-48.9) | 0.002 | 0.44(0.26-0.74) |  |  | < 0.001 | 0.39(0.23-0.67) |  |  |  |  |  |  |
| Normal (≤ 16.3) | < 0.001 | 0.25(0.15-0.43) |  |  | < 0.001 | 0.23(0.13-0.40) |  |  |  |  |  |  |
| CYFRA21-1 | 0.085 |  |  |  |  |  | 0.462 |  |  |  |  |  |
| 3-Fold(＞9.9) |  | 1 |  |  |  |  |  | 1 |  |  |  |  |
| Normal to 3-Fold (3.3-9.9) | 0.114 | 0.79(0.58-1.06) |  |  |  |  | 0.568 | 0.91(0.66-1.26) |  |  |  |  |
| Normal (≤ 3.3) | 0.061 | 0.64(0.40-1.02) |  |  |  |  | 0.348 | 0.79(0.49-1.29) |  |  |  |  |
| CA19-9 | 0.027 |  |  |  |  |  |  |  | 0.027 |  |  |  |
| 3-Fold(＞81) |  | 1 |  |  |  |  |  |  |  | 1 |  |  |
| Normal to 3-Fold (27-81) | 0.501 | 0.82(0.46-1.47) |  |  |  |  |  |  | 0.34 | 0.75(0.41-1.36) |  |  |
| Normal (≤ 27.0) | 0.038 | 0.56(0.32-0.97) |  |  |  |  |  |  | 0.013 | 0.48(0.27-0.86) |  |  |
| CA125 | 0.027 |  |  |  |  |  |  |  |  |  | < 0.001 |  |
| 3-Fold(＞105) |  | 1 |  |  |  |  |  |  |  |  |  | 1 |
| Normal to 3-Fold (35-105) | 0.454 | 0.85(0.57-1.29) |  |  |  |  |  |  |  |  | 0.055 | 0.65(0.42-1.01) |
| Normal (≤ 35.0) | 0.045 | 0.65(0.42-0.99) |  |  |  |  |  |  |  |  | < 0.001 | 0.43(0.27-0.67) |
| **Overall survival** |  |  |  |  |  |  |  |  |  |  |  |  |
| Age | 0.608 |  | 0.162 |  | 0.187 |  | 0.162 |  | 0.124 |  | 0.159 |  |
| Old ( ≥ 65) |  | 1 |  | 1 |  | 1 |  | 1 |  | 1 |  | 1 |
| Young (< 65) |  | 0.92(0.68-1.26) |  | 0.78(0.56-1.10) |  | 0.80(0.57-1.12) |  | 0.78(0.56-1.10) |  | 0.76(0.54-1.08) |  | 0.78(0.55-1.10) |
| Sex | 0.232 |  | 0.179 |  | 0.074 |  | 0.189 |  | 0.033 |  | 0.091 |  |
| Female |  | 1 |  | 1 |  | 1 |  | 1 |  | 1 |  | 1 |
| Male |  | 1.31(0.84-2.04) |  | 1.41(0.85-2.33) |  | 1.60(0.96-2.67) |  | 1.40(0.85-2.32) |  | 1.74(1.05-2.90) |  | 1.54(0.93-2.53) |
| ECOG PS | < 0.001 |  | < 0.001 |  | < 0.001 |  | < 0.001 |  | < 0.001 |  | < 0.001 |  |
| 0-1 |  | 1 |  | 1 |  | 1 |  | 1 |  | 1 |  | 1 |
| 2 |  | 3.52(2.40-5.16) |  | 3.29(2.19-4.94) |  | 3.53(2.36-5.29) |  | 3.35(2.23-5.04) |  | 3.61(2.38-5.47) |  | 3.80(2.50-5.78) |
| Smoke history | 0.589 |  | 0.964 |  | 0.611 |  | 0.902 |  | 0.67 |  | 0.722 |  |
| No |  | 1 |  | 1 |  | 1 |  | 1 |  | 1 |  | 1 |
| Yes |  | 1.09(0.80-1.49) |  | 1.01(0.71-1.43) |  | 1.10(0.77-1.56) |  | 1.02(0.72-1.45) |  | 0.93(0.65-1.32) |  | 0.94( 0.66-1.34) |
| Histological type | 0.009 |  | < 0.001 |  | < 0.001 |  | 0.024 |  | < 0.001 |  | < 0.001 |  |
| LUAD |  | 1 |  | 1 |  | 1 |  | 1 |  | 1 |  | 1 |
| LUSC | 0.005 | 1.60(1.15-2.22) | < 0.001 | 2.02(1.39-2.93) | < 0.001 | 1.83(1.28-2.63) | 0.008 | 1.63(1.13-2.35) | < 0.001 | 1.85(1.29-2.64) | < 0.001 | 1.87(1.30-2.67) |
| Other_NSCLC | 0.631 | 0.84(0.41-1.73) | 0.738 | 0.88(0.42-1.85) | 0.928 | 0.97(0.46-2.02) | 0.832 | 0.92(0.44-1.95) | 0.689 | 0.86(0.40-1.83) | 0.694 | 0.86(0.40-1.83) |
| Stage | 0.884 |  | 0.317 |  | 0.206 |  | 0.422 |  | 0.413 |  | 0.504 |  |
| IIIB |  | 1 |  | 1 |  | 1 |  | 1 |  | 1 |  | 1 |
| IIIC | 0.205 | 1.93(0.70-5.35) | 0.158 | 2.11(0.74-5.98) | 0.146 | 2.20(0.76-6.36) | 0.321 | 1.70(0.60-4.85) | 0.24 | 1.86(0.66-5.26) | 0.238 | 1.87(0.66-5.28) |
| IV | 0.92 | 1.04(0.49-2.22) | 0.402 | 1.40(0.64-3.09) | 0.231 | 1.63(0.73-3.65) | 0.489 | 1.32(0.60-2.94) | 0.467 | 1.34(0.61-2.93) | 0.585 | 1.25(0.57-2.74) |
| PD-L1 expression | 0.396 |  | 0.951 |  | 0.929 |  | 0.734 |  | 0.449 |  | 0.999 |  |
| < 1% |  | 1 |  | 1 |  | 1 |  | 1 |  | 1 |  | 1 |
| ≥ 1% |  | 0.82(0.51-1.31) |  | 0.98(0.60-1.61) |  | 0.98(0.60-1.60) |  | 1.09(0.66-1.79) |  | 1.22(0.73-2.04) |  | 1.00(0.61-1.64) |
| CEA | 0.435 |  | 0.039 |  |  |  |  |  |  |  |  |  |
| 3-Fold(＞15) |  | 1 |  | 1 |  |  |  |  |  |  |  |  |
| Normal to 3-Fold (5-15) | 0.792 | 0.95(0.64-1.41) | 0.497 | 0.87(0.58-1.31) |  |  |  |  |  |  |  |  |
| Normal (≤ 5.0) | 0.181 | 0.78(0.55-1.12) | 0.026 | 0.64(0.43-0.95) |  |  |  |  |  |  |  |  |
| NSE | < 0.001 |  |  |  | < 0.001 |  |  |  |  |  |  |  |
| 3-Fold(＞48.9) |  | 1 |  |  |  | 1 |  |  |  |  |  |  |
| Normal to 3-Fold (16.3-48.9) | < 0.001 | 0.38(0.22-0.63) |  |  | < 0.001 | 0.31(0.18-0.53) |  |  |  |  |  |  |
| Normal (≤ 16.3) | < 0.001 | 0.16(0.09-0.29) |  |  | < 0.001 | 0.14(0.08-0.25) |  |  |  |  |  |  |
| CYFRA21-1 | 0.026 |  |  |  |  |  | 0.032 |  |  |  |  |  |
| 3-Fold(＞9.9) |  | 1 |  |  |  |  |  | 1 |  |  |  |  |
| Normal to 3-Fold (3.3-9.9) | 0.039 | 0.71(0.51-0.98) |  |  |  |  | 0.133 | 0.77(0.54-1.08) |  |  |  |  |
| Normal (≤ 3.3) | 0.004 | 0.43(0.24-0.76) |  |  |  |  | 0.018 | 0.49(0.28-0.89) |  |  |  |  |
| CA19-9 | 0.038 |  |  |  |  |  |  |  | < 0.001 |  |  |  |
| 3-Flod(＞81) |  | 1 |  |  |  |  |  |  |  | 1 |  |  |
| Normal to 3-Fold (27-81) | 0.201 | 0.67(0.37-1.23) |  |  |  |  |  |  | 0.066 | 0.56(0.30-1.04) |  |  |
| Normal (≤ 27.0) | 0.004 | 0.44(0.25-0.77) |  |  |  |  |  |  | < 0.001 | 0.33(0.18-0.60) |  |  |
| CA125 | 0.041 |  |  |  |  |  |  |  |  |  | < 0.001 |  |
| 3-Fold(＞105) |  | 1 |  |  |  |  |  |  |  |  |  | 1 |
| Normal to 3-Fold (35-105) | 0.605 | 0.89(0.56-1.40) |  |  |  |  |  |  |  |  | 0.059 | 0.63(0.39-1.02) |
| Normal (≤ 35.0) | 0.039 | 0.61(0.38-0.98) |  |  |  |  |  |  |  |  | < 0.001 | 0.41(0.25-0.68) |

| **Table S4.** Prognostic factors for progression-free survival and overall survival in patients with advanced non-small cell lung cancer who received combination therapy. | | | | | | | | | | | | |
| --- | --- | --- | --- | --- | --- | --- | --- | --- | --- | --- | --- | --- |
| Covariate | Univariate analysis | | Multivariate analysis (CEA) | | Multivariate analysis (NSE) | | Multivariate analysis (CYFRA21-1) | | Multivariate analysis (CA19-9) | | Multivariate analysis (CA125) | |
|  | *P* | HR (95% CI) | *P* | HR (95% CI) | *P* | HR (95% CI) | *P* | HR (95% CI) | *P* | HR (95% CI) | *P* | HR (95% CI) |
| **Progression-free survival** |  |  |  |  |  |  |  |  |  |  |  |  |
| Age | 0.571 |  | 0.905 |  | 0.985 |  | 0.858 |  | 0.592 |  | 0.488 |  |
| Old ( ≥ 65) |  | 1 |  | 1 |  | 1 |  | 1 |  | 1 |  | 1 |
| Young (< 65) |  | 0.94(0.75-1.17) |  | 0.99(0.78-1.25) |  | 1.00(0.79-1.27) |  | 0.99(0.77-1.24) |  | 0.94(0.74-1.19) |  | 0.92(0.72-1.16) |
| Sex | 0.893 |  | 0.409 |  | 0.091 |  | 0.293 |  | 0.262 |  | 0.381 |  |
| Female |  | 1 |  | 1 |  | 1 |  | 1 |  | 1 |  | 1 |
| Male |  | 1.02(0.74-1.42) |  | 1.16(0.81-1.67) |  | 1.37(0.95-1.96) |  | 1.21(0.85-1.74) |  | 1.23(0.86-1.76) |  | 1.18(0.82-1.69) |
| ECOG PS | < 0.001 |  | < 0.001 |  | < 0.001 |  | < 0.001 |  | < 0.001 |  | < 0.001 |  |
| 0-1 |  | 1 |  | 1 |  | 1 |  | 1 |  | 1 |  | 1 |
| 2 |  | 2.80(1.90-4.14) |  | 2.24(1.49-3.37) |  | 2.61(1.72-3.94) |  | 2.77(1.82-4.19) |  | 2.53(1.67-3.82) |  | 2.64(1.74-4.00) |
| Smoke history | 0.475 |  | 0.083 |  | 0.924 |  | 0.549 |  | 0.507 |  | 0.399 |  |
| No |  | 1 |  | 1 |  | 1 |  | 1 |  | 1 |  | 1 |
| Yes |  | 0.92(0.74-1.15) |  | 0.79(0.61-1.03) |  | 0.99(0.76-1.28) |  | 0.93(0.71-1.19) |  | 0.92(0.71-1.18) |  | 0.90( 0.70-1.16) |
| Histological type | < 0.001 |  | < 0.001 |  | < 0.001 |  | 0.008 |  | < 0.001 |  | 0.001 |  |
| LUAD |  | 1 |  | 1 |  | 1 |  | 1 |  | 1 |  | 1 |
| LUSC | 0.138 | 1.18(0.95-1.48) | 0.002 | 1.46(1.15-1.85) | 0.036 | 1.28(1.01-1.62) | 0.373 | 1.11(0.88-1.42) | 0.067 | 1.24(0.99-1.57) | 0.026 | 1.31(0.15-0.63) |
| Other_NSCLC | < 0.001 | 0.29(0.14-0.59) | 0.003 | 0.34(0.16-0.70) | < 0.001 | 0.25(0.12-0.51) | 0.002 | 0.32(0.15-0.65) | < 0.001 | 0.28(0.14-0.57) | 0.001 | 0.30(1.03-1.65) |
| Stage | < 0.001 |  | < 0.001 |  | < 0.001 |  | < 0.001 |  | < 0.001 |  | < 0.001 |  |
| IIIB |  | 1 |  | 1 |  | 1 |  | 1 |  | 1 |  | 1 |
| IIIC | < 0.001 | 4.77(2.60-8.73) | < 0.001 | 4.94(2.62-9.33) | < 0.001 | 3.67(1.94-6.95) | < 0.001 | 3.64(1.91-6.95) | < 0.001 | 3.87(2.04-7.35) | < 0.001 | 4.10(2.16-7.79) |
| IV | 0.021 | 1.46(1.06-2.00) | 0.045 | 1.40(1.01-1.95) | 0.035 | 1.42(1.03-1.98) | 0.015 | 1.50(1.08-2.09) | 0.028 | 1.44(1.04-2.02) | 0.029 | 1.44(1.04-2.01) |
| PD-L1 expression | 0.293 |  | 0.699 |  | 0.765 |  | 0.557 |  | 0.329 |  | 0.237 |  |
| < 1% |  | 1 |  | 1 |  | 1 |  | 1 |  | 1 |  | 1 |
| ≥ 1% |  | 0.88(0.69-1.12) |  | 0.95(0.73-1.23) |  | 0.96(0.74-1.25) |  | 0.92(0.71-1.20) |  | 0.88(0.68-1.14) |  | 0.86(0.66-1.11) |
| CEA | < 0.001 |  | < 0.001 |  |  |  |  |  |  |  |  |  |
| 3-Fold(＞15) |  | 1 |  | 1 |  |  |  |  |  |  |  |  |
| Normal to 3-Fold (5-15) | 0.103 | 0.79(0.60-1.05) | 0.132 | 0.80(0.60-1.07) |  |  |  |  |  |  |  |  |
| Normal (≤ 5.0) | < 0.001 | 0.40(0.30-0.52) | < 0.001 | 0.36(0.27-0.48) |  |  |  |  |  |  |  |  |
| NSE | < 0.001 |  |  |  | < 0.001 |  |  |  |  |  |  |  |
| 3-Fold(＞48.9) |  | 1 |  |  |  | 1 |  |  |  |  |  |  |
| Normal to 3-Fold (16.3-48.9) | < 0.001 | 0.37(0.24-0.56) |  |  | < 0.001 | 0.35(0.23-0.54) |  |  |  |  |  |  |
| Normal (≤ 16.3) | < 0.001 | 0.27(0.17-0.41) |  |  | < 0.001 | 0.25(0.16-0.39) |  |  |  |  |  |  |
| CYFRA21-1 | < 0.001 |  |  |  |  |  | < 0.001 |  |  |  |  |  |
| 3-Fold(＞9.9) |  | 1 |  |  |  |  |  | 1 |  |  |  |  |
| Normal to 3-Fold (3.3-9.9) | < 0.001 | 0.65(0.52-0.82) |  |  |  |  | 0.002 | 0.69(0.54-0.88) |  |  |  |  |
| Normal (≤ 3.3) | < 0.001 | 0.40(0.27-0.59) |  |  |  |  | < 0.001 | 0.46(0.31-0.68) |  |  |  |  |
| CA19-9 | 0.007 |  |  |  |  |  |  |  | 0.012 |  |  |  |
| 3-Fold(＞81) |  | 1 |  |  |  |  |  |  |  | 1 |  |  |
| Normal to 3-Fold (27-81) | 0.056 | 0.67(0.45-1.01) |  |  |  |  |  |  | 0.073 | 0.69(0.45-1.04) |  |  |
| Normal (≤ 27.0) | 0.003 | 0.56(0.39-0.82) |  |  |  |  |  |  | 0.005 | 0.58(0.39-0.85) |  |  |
| CA125 | < 0.001 |  |  |  |  |  |  |  |  |  | < 0.001 |  |
| 3-Fold(＞105) |  | 1 |  |  |  |  |  |  |  |  |  | 1 |
| Normal to 3-Fold (35-105) | < 0.001 | 0.38(0.27-0.54) |  |  |  |  |  |  |  |  | < 0.001 | 0.36(0.25-0.51) |
| Normal (≤ 35.0) | < 0.001 | 0.32(0.23-0.46) |  |  |  |  |  |  |  |  | < 0.001 | 0.32(0.22-0.46) |
| **Overall survival** |  |  |  |  |  |  |  |  |  |  |  |  |
| Age | 0.004 |  | 0.018 |  | 0.046 |  | 0.03 |  | 0.009 |  | 0.005 |  |
| Old ( ≥ 65) |  | 1 |  | 1 |  | 1 |  | 1 |  | 1 |  | 1 |
| Young (< 65) |  | 0.70(0.54-0.89) |  | 0.73(0.56-0.95) |  | 0.76(0.58-0.99) |  | 0.75(0.58-0.97) |  | 0.70(0.54-0.92) |  | 0.68(0.53-0.89) |
| Sex | 0.224 |  | 0.193 |  | 0.123 |  | 0.124 |  | 0.126 |  | 0.246 |  |
| Female |  | 1 |  | 1 |  | 1 |  | 1 |  | 1 |  | 1 |
| Male |  | 1.26(0.87-1.83) |  | 1.32(0.87-2.02) |  | 1.39(0.91-2.12) |  | 1.39(0.91-2.13) |  | 1.39(0.91-2.11) |  | 1.28(0.84-1.96) |
| ECOG PS | < 0.001 |  | < 0.001 |  | < 0.001 |  | < 0.001 |  | < 0.001 |  | < 0.001 |  |
| 0-1 |  | 1 |  | 1 |  | 1 |  | 1 |  | 1 |  | 1 |
| 2 |  | 3.25(2.20-4.81) |  | 2.69(1.78-4.05) |  | 3.05(2.01-4.62) |  | 3.28(2.17-4.97) |  | 3.06(2.03-4.62) |  | 3.08(2.03-4.66) |
| Smoke history | 0.492 |  | 0.212 |  | 0.65 |  | 0.821 |  | 0.667 |  | 0.87 |  |
| No |  | 1 |  | 1 |  | 1 |  | 1 |  | 1 |  | 1 |
| Yes |  | 1.09(0.85-1.39) |  | 0.83(0.63-1.11) |  | 1.07(0.80-1.42) |  | 0.97(0.73-1.28) |  | 0.94(0.71-1.25) |  | 0.98( 0.74-1.30) |
| Histological type | 0.041 |  | 0.018 |  | 0.024 |  | 0.048 |  | 0.031 |  | 0.037 |  |
| LUAD |  | 1 |  | 1 |  | 1 |  | 1 |  | 1 |  | 1 |
| LUSC | 0.16 | 1.20(0.93-4.53) | 0.003 | 1.49(1.14-1.95) | 0.162 | 1.20(0.93-1.55) | 0.62 | 1.07(0.82-1.39) | 0.151 | 1.21(0.93-1.56) | 0.089 | 1.25(0.97-1.62) |
| Other_NSCLC | 0.022 | 0.43(0.21-0.88) | 0.061 | 0.49(0.24-1.03) | 0.006 | 0.37(0.18-0.75) | 0.044 | 0.47(0.23-0.98) | 0.011 | 0.39(0.19-0.81) | 0.025 | 0.44(0.21-0.90) |
| Stage | 0.884 |  | 0.041 |  | 0.311 |  | 0.222 |  | 0.413 |  | 0.218 |  |
| IIIB |  | 1 |  | 1 |  | 1 |  | 1 |  | 1 |  | 1 |
| IIIC | 0.019 | 2.20(1.14-4.23) | 0.03 | 2.15(1.08-4.29) | 0.278 | 1.47(0.73-2.95) | 0.214 | 1.56(0.77-3.17) | 0.133 | 1.71(0.85-3.46) | 0.081 | 1.86(0.93-3.73) |
| IV | 0.139 | 1.31(0.92-1.86) | 0.459 | 1.15(0.80-1.66) | 0.381 | 1.18(0.82-1.70) | 0.234 | 1.25(0.87-1.80) | 0.349 | 1.19(0.83-1.72) | 0.329 | 1.20(0.83-1.73) |
| PD-L1 expression | 0.004 |  | 0.195 |  | 0.115 |  | 0.058 |  | 0.026 |  | 0.011 |  |
| < 1% |  | 1 |  | 1 |  | 1 |  | 1 |  | 1 |  | 1 |
| ≥ 1% |  | 0.68(0.52-0.88) |  | 0.83(0.63- 1.10) |  | 0.80(0.60-1.06) |  | 0.76(0.58-1.01) |  | 0.73(0.55-0.96) |  | 0.69(0.52-0.92) |
| CEA | < 0.001 |  | < 0.001 |  |  |  |  |  |  |  |  |  |
| 3-Fold(＞15) |  | 1 |  | 1 |  |  |  |  |  |  |  |  |
| Normal to 3-Fold (5-15) | 0.27 | 0.85(0.63-1.14) | 0.193 | 0.81(0.59-1.11) |  |  |  |  |  |  |  |  |
| Normal (≤ 5.0) | < 0.001 | 0.34(0.25-0.47) | < 0.001 | 0.32(0.23-0.44) |  |  |  |  |  |  |  |  |
| NSE | < 0.001 |  |  |  | < 0.001 |  |  |  |  |  |  |  |
| 3-Fold(＞48.9) |  | 1 |  |  |  | 1 |  |  |  |  |  |  |
| Normal to 3-Fold (16.3-48.9) | < 0.001 | 0.38(0.24-0.60) |  |  | 0.001 | 0.47(0.30-0.74) |  |  |  |  |  |  |
| Normal (≤ 16.3) | < 0.001 | 0.31(0.20-0.49) |  |  | < 0.001 | 0.36(0.23-0.58) |  |  |  |  |  |  |
| CYFRA21-1 | < 0.001 |  |  |  |  |  | < 0.001 |  |  |  |  |  |
| 3-Fold(＞9.9) |  | 1 |  |  |  |  |  | 1 |  |  |  |  |
| Normal to 3-Fold (3.3-9.9) | < 0.001 | 0.61(0.47-0.79) |  |  |  |  | 0.002 | 0.66(0.51-0.86) |  |  |  |  |
| Normal (≤ 3.3) | < 0.001 | 0.35(0.22-0.55) |  |  |  |  | < 0.001 | 0.41(0.26-0.66) |  |  |  |  |
| CA19-9 | 0.001 |  |  |  |  |  |  |  | < 0.001 |  |  |  |
| 3-Flod(＞81) |  | 1 |  |  |  |  |  |  |  | 1 |  |  |
| Normal to 3-Fold (27-81) | 0.018 | 0.59(0.38-0.91) |  |  |  |  |  |  | 0.009 | 0.55(0.35-0.86) |  |  |
| Normal (≤ 27.0) | < 0.001 | 0.46(0.31-0.69) |  |  |  |  |  |  | < 0.001 | 0.45(0.30-0.68) |  |  |
| CA125 | < 0.001 |  |  |  |  |  |  |  |  |  | < 0.001 |  |
| 3-Fold(＞105) |  | 1 |  |  |  |  |  |  |  |  |  | 1 |
| Normal to 3-Fold (35-105) | < 0.001 | 0.33(0.23-0.48) |  |  |  |  |  |  |  |  | < 0.001 | 0.63(0.20-0.44) |
| Normal (≤ 35.0) | < 0.001 | 0.29(0.20-0.43) |  |  |  |  |  |  |  |  | < 0.001 | 0.28(0.19-0.42) |
